# Thiol isomerase ERp18 enhances platelet activation and arterial thrombosis

**DOI:** 10.1016/j.rpth.2025.102706

**Published:** 2025-02-27

**Authors:** Chao He, Aizhen Yang, Keyu Lv, Yuxin Zhang, Zhenzhen Zhao, Yi Lu, Chao Fang, Yue Han, Depei Wu, Miao Jiang, Jingyu Zhang, Yi Wu

**Affiliations:** 1 Cyrus Tang Medical Institute, Collaborative Innovation Center of Hematology, State Key Laboratory of Radiation Medicine and Prevention, The Fourth Affiliated Hospital of Soochow University, Suzhou, China; 2 Department of Hematology, The Second Hospital of Hebei Medical University, Hebei Key Laboratory of Hematology, Shijiazhuang, China; 3 Department of Pharmacology, Tongji Medical College of Huazhong University of Science and Technology, Wuhan, China; 4 Hunan Sinozex Biosciences Co, Ltd, Changsha, China; 5 Department of Hematology, National Clinical Research Center for Hematologic Diseases, Jiangsu Institute of Hematology, Institute of Blood and Marrow Transplantation, Soochow University, Suzhou, China

**Keywords:** ERp18, integrin α_IIb_β_3_, platelets, redox regulation, thrombosis

## Abstract

**Background:**

Thiol isomerases regulate the thiol-disulfide exchange of functional proteins in cells. Using genetically modified mouse models and inhibitors, we and others demonstrated that 7 thiol isomerases (ERp57, protein diisulfide isomerase, ERp72, ERp46, ERp5, TMX4, and TMX1) participate in thrombosis. There are 21 thiol isomerases in mammals, but whether other enzymes of this family also contribute to thrombosis remains unknown.

**Objectives:**

Investigate whether and how ERp18 participates in arterial thrombosis.

**Methods:**

ERp18 knockout mice and arterial thrombosis models were used to determine the role of ERp18 in thrombosis. Platelets from ERp18 knockout mice were used to detect aggregation, activation, spreading, and clot retraction. Finally, flow cytometry and immunoprecipitation were used to detect the binding between ERp18 and α_IIb_β_3_.

**Results:**

The mice lacking ERp18 exhibited a prolonged tail bleeding time and decreased platelet thrombus formation in FeCl_3_-induced carotid arterial injury and laser-induced cremaster artery injury models. ERp18 deficiency inhibited platelet aggregation, adenosine triphosphate release, integrin α_IIb_β_3_ activation, P-selectin expression, platelet adhesion, as well as clot retraction. Flow cytometry and coimmunoprecipitation analyses revealed that ERp18 binds to the platelet surface via interaction with integrin α_IIb_β_3_. Moreover, the ERp18 protein promoted the binding of integrin α_IIb_β_3_ to fibrinogen and platelet aggregation. Furthermore, the recombinant ERp18 protein exhibited reductase activity and cleaved integrin α_IIb_β_3_ disulfides.

**Conclusion:**

ERp18 participates in platelet activation and thrombosis. Its function is, at least in part, through the regulation of integrin α_IIb_β_3_ function. This finding expands our understanding of the role of thiol isomerases in the redox regulation of thrombosis and platelet function.

## Essentials

• Seven isomerases participate in arterial thrombosis, while other isomerases remain unknown.
• We investigated whether and how ERp18 participates in arterial thrombosis.
• ERp18 promotes platelet activation and thrombosis.
• Its function is, at least in part, through the regulation of integrin α_IIb_β_3_ function.

## 1 Introduction

Thiol isomerases are a group of oxidoreductases in the endoplasmic reticulum that catalyze the correct disulfide bond formation during the protein folding process [1]. In recent decades, thiol isomerases have been found to play an important role in constituting “off-on” redox switches of thrombosis [2]. Using genetically modified mouse models and inhibitors, we and others have found that ERp57, protein diisulfide isomerase (PDI), ERp72, ERp5, ERp46, and TMX4 are critical in supporting platelet activation and thrombus formation [[3], [4], [5], [6], [7], [8], [9], [10]]. However, the type I transmembrane thiol isomerase TMX1 has an opposite role and inhibits these processes [11]. These positive and negative thiol isomerases form an essential redox network balancing thrombosis. All of these thiol isomerases contain 1 to 3 Cys-X-X-Cys (CXXC) active sites [12], and their catalytic activity is required for regulating platelet activation and thiol-disulfide exchange in integrin α_IIb_β_3_, suggesting the importance of CXXC active site-containing thiol isomerases in thrombosis [[13], [14], [15]]. With 21 thiol isomerases in mammals, it is crucial to determine whether other thiol isomerases containing the CXXC motif also contribute to thrombosis and hemostasis [16].

ERp18, first characterized in 2003 by Alanen et al. [17], contains a single catalytic domain featuring a CGAC active motif. It has been shown that ERp18 regulates the structure and function of substrates by catalyzing the reduction and oxidation of allosteric disulfide bonds [17,18]; thus, ERp18 has a role in cellular functions. For example, ERp18 regulates ATF6α activation during the unfolded protein response [19]. Although ERp18 is expressed at a level comparable to that of PDI in megakaryocytes [16], its biological role in the regulation of thrombosis and hemostasis has never been characterized. In this study, we generated a new model of whole-body ERp18 knockout (KO) mice to investigate the role of ERp18 in platelet function and arterial thrombosis. We found that ERp18 deficiency prolonged the bleeding time and decreased platelet activation and platelet thrombus formation *in vivo*. ERp18 bound to integrin α_IIb_β_3_ and reduced its disulfides, which was associated with integrin activation. This study provides the first genetic evidence demonstrating that ERp18 is important for supporting platelet function and arterial thrombosis via its activation of integrin α_IIb_β_3_. Thus, the novel role of ERp18 in platelet function expands our understanding of the contribution of thiol isomerases to hemostasis and thrombosis.

## 2 Methods

### 2.1 Reagents

Anti-ERp18 antibodies were obtained from Abcam (ab134938) and Novus Biologicals (NBP2-93457). PE-conjugated anti–P-selectin antibody (MA5-28649) and anti-mouse CD61 (12-0611-81) were acquired from eBioscience Inc. PE-labeled JON/A antibody (M023-2), anti-mouse glycoprotein (GP) Ibα (M040-2), GPVI (M011-1), anti-CD42b (R300), and anti-mouse CD42c (X488) were purchased from Emfret Analytics. Anti-CD31 (ab182981) was obtained from Abcam. Anti–β-actin antibody (GTX109639) was purchased from GeneTex. GAPDH (60004-1-lg) antibody was acquired from Proteintech. Thrombin, U46619, and luciferin/luciferase reagents were from Chrono-Log Corporation. IRDye 800CW goat anti-rabbit IgG (926-32211) secondary antibody was bought from LI-COR Biosciences. Polymerase chain reaction (PCR) Green Taq Mix (P131-01), GelRed (GR501-01), DNA marker (MD104-02), Trizol (R401-01), and HiScript II Q Select RT SuperMix (R233-01) were purchased from Vazyme. Protein marker (26616) was bought from Thermo Fisher Scientific. Fluorescein isothiocyanate (FITC)-labeled annexin V (C1062L) and Fluo-4 AM (S1060) were purchased from Beyotime. 4′,6-diamidino-2-phenylindole (0100-20) was purchased from SouthernBiotech. The fluorescent thrombin substrate (86197) was purchased from Stago. 3-(N-maleimido-propionyl) biocytin (*MPB*), and fibrinogen (F3879) were purchased from Sigma.

### 2.2 Mice

ERp18-floxed mice were generated by Cyagen using the clustered regularly interspaced short palindromic repeats/Cas9 system. CAG-Cre mice were purchased from Gempharmatech. To produce whole-body ERp18-KO mice, the ERp18^flox/flox^ (ERp18^fl/fl^) mice were crossed with CAG-Cre mice. Heterozygous KO mice were mated, and offspring were identified by genotyping using the following primers: CAG-Cre-F 5′-CCTGCTGTCCATTCCTTATTCCATA-3′, CAG-Cre-R 5′-ATATCCCCTTGTTCCCTTTCTGC-3′; and ERp18-F 5′-CCCAGAAGTTCTAGTGACTGTAGG-3′, ERp18-R 5′-CCACAGATTTGGGCTTTAAAAGTAGG-3′. β_3_ integrin-null mice on a C57BL/6 background, along with matched C57BL/6 mice, were used as previously described [7]. In this study, the mice were fed standard rodent chow and water *ad libitum* and were maintained under climate-controlled conditions in a 12-hour light/dark cycle in a pathogen-free facility. The health status of the animals was monitored following the guidelines of the Institutional Animal Care and Use Committee (Soochow University) with an approved animal protocol. Total RNA was extracted from major organs of mice to verify the KO efficiency. In the PCR reactions, complementary DNA was obtained by reverse transcription and amplified using the following specific primers: ERp18-F 5′-TCTTGGTGTGGAGCCTGCAAAG-3′, ERp18-R 5′-CATCAGGGCTGAAGTCTTCATCC-3′; and β-actin forward primer, 5′-GTGCTATGTTGCTCTAGACTTCG-3′, reverse primer, 5′-ATGCCACAGGATTCCATACC-3′.

### 2.3 Complete blood counts of mice

Mice were anesthetized with sodium pentobarbital (65 mg/kg) via intraperitoneal injection, and blood was collected through retro-orbital bleeding using EDTA-2Na as an anticoagulant. Complete blood counts were measured using a Sysmex Coulter Counter (XT2000-iV).

### 2.4 Bleeding time analysis

A razor blade was used to cut the mouse tail 4.5 mm from the tip, which was then immersed in 15 mL of 0.9% NaCl at 37 °C. After the bleeding stopped for at least 15 seconds, the bleeding times were determined. The assay was halted if bleeding lasted longer than 15 minutes, and the bleeding time was recorded as 15 minutes.

### 2.5 Confocal fluorescence microscopy analysis

Freshly isolated mouse aortas from mice were placed into a 12-well plate and fixed with 4% paraformaldehyde for 2 hours at room temperature. The fixed tissues were washed and dehydrated in 20% sucrose solution for 24 hours at 4 °C. Each tissue was then immersed in a Tissue-Tek optimum cutting temperature Compound (4583; from SAKURA Inc) at room temperature for 30 minutes, transferred to a tinfoil cryomold, filled with optimum cutting temperature Compound, and frozen at −20 °C until solidified. Tissue sections (8 μm) were made on microscopy slides (Citotest Scientific). The sections were fixed with cold acetone for 20 minutes and washed 3 times with 1× phosphate-buffered saline (PBS). After blocking with 1% bovine serum albumin at room temperature for 1 hour, the sections were stained with anti-CD31 (Abcam) and anti-ERp18 (Novus Biologicals) antibodies, as well as 4′,6-diamidino-2-phenylindole (SouthernBiotech). The sections were imaged using a laser confocal microscope (Olympus).

### 2.6 FeCl_3_-induced carotid artery injury

FeCl_3_-induced carotid artery injury was performed as previously described [20]. Mice were anesthetized with sodium pentobarbital 65 mg/kg via intraperitoneal injection. The left carotid artery was surgically exposed by blunt dissection through a midline incision in the neck. Whatman filter paper soaked in 5% FeCl_3_ was applied under and above the exposed artery for 90 seconds. Following the removal of the filter paper, the artery was cleaned with PBS, and an imaging ultrasound gel (MS400-0090; VisualSonics) was applied to allow Doppler monitoring. A small animal blood flow transducer (MS400, 18-38 MHz; VisualSonics) and the color Doppler mode of the VisualSonics Vevo model 2100 flowmeter were used to identify the artery. The transducer was fixed in place using the Vevo Imaging Station (Integrated Rail System). The temperature of mice was maintained at 37 °C. Using the 2-dimensional imaging Doppler mode, the baseline systolic and diastolic blood velocity (mm/s) was measured. The occlusion time was defined as the period of time between initiation of arterial injury and complete occlusion of blood flow. Occlusion is considered stable when the flow has been completely stopped for at least 5 minutes.

### 2.7 FeCl_3_-induced platelet accumulation and thrombosis of mesenteric artery

To deplete platelets in ERp18-KO mice, anti-CD42b monoclonal antibodies (2 μg/g weight, Emfret Analytics) were injected through the tail vein [21]. Blood from wild-type (WT) mice was collected, and washed platelets were prepared and resuspended in sterile PBS. Platelet-depleted ERp18-KO mice were replenished with washed WT platelets at counts comparable to WT and ERp18-KO mice. To evaluate platelet thrombus formation, an anti-CD42c antibody (0.1 μg/g weight) was injected into mice through the tail vein, as we previously described [8]. Briefly, the mesentery was exteriorized through a midline abdominal incision, and the artery was visualized under a Lecia DM 2000 fluorescence microscope. To induce vessel injury, a patch of filter paper soaked in 5% FeCl_3_ was placed above the exposed artery for 2 minutes. After the removal of the filter paper, platelet accumulation was observed and photographed. The images were analyzed by ImageJ software (National Institutes of Health).

### 2.8 Laser-induced cremaster arterial injury

Laser injury-induced thrombosis in the cremaster arterioles was performed as previously described [22]. In brief, mice were anesthetized by intraperitoneal injection of sodium pentobarbital at 65 mg/kg body weight. The cremaster vasculature of male WT mice or ERp18-KO mice was exposed and immersed in bicarbonate buffer balanced with CO_2_. After 5 minutes, thrombus formation was induced by a nitrogen laser pulse that injured the cremaster arteriole endothelium. Platelets and fibrin were visualized using an anti-CD41 antibody conjugated with Alexa Fluor 488 (A10235; Invitrogen) and an antifibrin antibody conjugated with Alexa Fluor 647, respectively. Images were captured using high-speed intravital microscopy and analyzed with SlideBook 6.0 (Intelligent Imaging Innovations). At least 30 thrombi were collected from 3 mice in each group. The medium fluorescence intensity and area under the curve for each thrombus were calculated and graphed.

### 2.9 Washed platelet preparation

Platelets were isolated from mouse or human blood as previously described [23,24]. For mouse platelet studies, blood was collected from the inferior vena cava using 120 mM trisodium citrate, 111 mM dextrose, and 80 mM citric acid as an anticoagulant and diluted in modified Tyrode’s buffer (137 mM NaCl, 5.6 mM glucose, 2.7 mM KCl, 1 mM MgCl_2_, 3.3 mM NaH_2_PO_4_, 20 mM 4-(2-hydroxyethyl)piperazine-1-ethanesulfonic acid, pH 7.4). Platelets were then pelleted by centrifugation at 800 rpm in the presence of prostaglandin E1 (LKT-P6956; Enzo Life Science) (1 μM), washed twice, and resuspended in modified Tyrode’s buffer.

### 2.10 Flow cytometric measurement, aggregation, and secretion studies of platelets

Flow cytometry studies of platelets were conducted with washed platelets [25]. To assess the binding of fibrinogen to platelets, purified fibrinogen was labeled using the FluoReporter FITC Protein Labeling Kit following the manufacturer’s protocol (F6434, Invitrogen). Briefly, 2 × 10^7^/mL washed human platelets or ERp18-KO mice platelets were preincubated with or without ERp18 protein at 37 °C for 10 minutes, followed by incubation with thrombin in the presence of 10 μg/mL FITC-conjugated fibrinogen. Platelets were fixed with 1% paraformaldehyde (PFA) in PBS buffer for 10 minutes, washed, and analyzed by flow cytometry. Aggregation studies were carried out using washed platelets [8]. Platelet count was measured using a Sysmex Coulter, and counts were adjusted to 200,000 to 250,000/μL for aggregation studies. Secretion of adenosine triphosphate (ATP) was monitored using the Chrono-Log Lumi-Aggregometer, as previously described [8].

### 2.11 Platelet adhesion assay

A platelet adhesion assay was performed using washed mouse platelets as described [26]. Briefly, coverslips were coated with 5 μg/mL fibrinogen overnight at 4 °C and then blocked with 2% fatty acid-free bovine serum albumin for 2 hours at room temperature. Washed platelets in Tyrode’s buffer or platelets treated with 0.014 U/mL thrombin were then seeded on coverslips and incubated at 37 °C for 15 minutes. Following washing and fixation with 4% PFA, platelets were stained with tetramethylrhodamine-conjugated phalloidin, supplemented with 0.1% Triton X-100 (T8200; Solarbio Life Science Inc) for 2 hours. Subsequently, samples were observed under a confocal microscope (Olympus FV3000) and photographed. The number and area of platelets in the image were calculated using ImageJ software.

### 2.12 Platelet clot retraction

Clot retraction was performed as described previously with some modifications [27]. Platelet-poor plasma (PPP) from WT mice was anticoagulated with 3.8% sodium citrate and combined with washed murine platelets to achieve a concentration of 2.5 × 10^8^/mL. The clot retraction process was initiated with 0.1 U/mL thrombin, and images were captured at increasing times. Two-dimensional clot size was measured using ImageJ software, and the percentage of clot size in relation to the initial suspension volume was determined.

### 2.13 Generation of recombinant human ERp18 WT protein (CGAC) and ERp18 mutant protein (SGAS)

Human ERp18 (UniProtKB: O95881; ERp18-WT) was expressed in *Escherichia coli* strain BL21 (DE3) pLysS and purified using a Ni Sepharose High-Performance column (GE Healthcare). The mutant recombinant ERp18 protein (ERp18-Mut) was generated by mutating cysteine residues of the active site of human ERp18 with serine residues (C66S and C69S) using the primer 5′-GATTATTCATA AATCCTGGAGTGGAGCTAGCAAAG-3′. DNA sequencing confirmed the correct base substitutions. The purified proteins were run on 10% sodium dodecyl sulfate (SDS)–polyacrylamide gel electrophoresis gels, and the purity was more than 85%, confirmed by Coomassie staining.

### 2.14 Labeling of free thiols in integrin α_IIb_β_3_ with MPB

A total of 1.5 μg of purified human integrin α_IIb_β_3_ (ab95130, Abcam) was incubated with 2 μM ERp18-WT or ERp18-Mut for 30 minutes at 37 °C. Thiols were labeled with 18 μM MPB in PBS (containing 1% SDS, 5 mM EDTA at pH 7.4) for 25 minutes at room temperature. After MPB labeling, reactions were incubated with or without 40 μM glutathione (Sangon Biotech Corporation) for 15 minutes. Blotting was performed using avidin-horseradish peroxidase, monoclonal anti-ERp18, and anti-β_3_ antibodies.

### 2.15 Coimmunoprecipitation of integrin α_IIb_β_3_ and ERp18

Immunoprecipitation was performed as previously described with minor modifications [28]. Briefly, platelets were lysed with NP-40 (N8032; Solarbio Life Science Inc) buffer (50 mM Tris-HCl, 150 mM NaCl, 1% NP-40, 5 mM EDTA, 1 mM Na_3_VO_4_, 1 mM phenylmethylsulfonyl fluoride, 2× protease inhibitor cocktail, leupeptin 10 μM, pH 7.4). Lysates were incubated with mouse anti-β_3_ antibody (2.5 μg/mL, SZ-21; was a gift from Dr Changgeng Ruan) with overnight rotation followed by protein G resin (L00209; GenScript Biotech Corporation) for 60 minutes at 4 °C. Western blotting was performed with goat anti-ERp18 antibody and monoclonal anti-β_3_ antibody (sc-365679, Santa Cruz Biotechnology). The polyvinylidene fluoride membranes were developed using IRDye 800CW-conjugated secondary antibodies diluted at 1:10,000 and imaged using an LI-COR Odyssey IR imaging system (LI-COR Biosciences).

### 2.16 Western blotting

Protein samples were separated by 8% to 12% SDS–polyacrylamide gel electrophoresis and then transferred to a polyvinylidene fluoride membrane. A 5% skim milk in Tris-buffered saline was used to block the membrane for 1 hour at room temperature. After washing with Tris-buffered saline, the membrane was incubated with primary antibodies overnight at 4 °C. Antibody binding was detected using IRDye 800CW-conjugated goat anti-rabbit IgG and visualized with an Odyssey IR imaging system.

### 2.17 Di-E-GSSG assay

As previously described [29], the dieosinediglutathione (Di-E-GSSG) assay was performed in an assay buffer (0.1 M potassium phosphate buffer, 2 mM EDTA, pH 7.0). ERp18-WT, ERp18-Mut (2 μM), and PBS as control were added to Di-E-GSSG (150 nM, Cayman Chemical) in the presence of dithiothreitol (D8220; Solarbio Life Science Inc) (5 μM) in a quartz cuvette. The increase in fluorescence was monitored at 545 nm with excitation at 525 nm.

### 2.18 Thrombin generation assay

Blood was collected from the inferior vena cava of mice with 3.2% sodium citrate. PPP, platelet-rich plasma, and plasma were obtained through a series of centrifugations. Subsequently, platelet-rich plasma, PPP, or plasma was added to a thrombin generation assay cuvette and triggered by 1.0 pM tissue factor, 5 pM tissue factor plus 4 μM phospholipids, or thrombin (0.1 U/mL) plus collagen (10 μg/mL). Thrombin generation was initiated by the automatic dispensation of fluorescent thrombin substrate containing calcium (Ca^2+^) and assessed in real-time by Ceveron Alpha thrombin generation assay (Technoclone).

### 2.19 Ca^2+^ mobilization

To analyze intracellular Ca^2+^ signals, washed mouse platelets from WT and ERp18-KO mice (5 × 10^7^/mL) were loaded with Fluo-4 AM (2 μM) at 37 °C for 30 minutes in the dark. After mixing with Tyrode’s buffer, thrombin (0.08 U/mL) was added along with CaCl_2_ (1 mM) to initiate the reaction. Fluorescence signals were detected using a Becton Dickinson flow cytometer and recorded for 3 minutes. The baseline signal was calculated as the average fluorescence intensity from the initial 20 seconds prior to thrombin stimulation. To control unwanted variations in baseline fluorescence, the signal at each time point was normalized to the baseline value of each sample. Alternatively, fluorescence in platelets was measured using a microplate reader at excitation/emission wavelengths = 494/516 nm. The fluorescence intensity was calculated by subtracting the fluorescence intensity of thrombin-stimulated platelets from that of untreated platelets.

### 2.20 Phosphatidylserine exposure on platelets

A thrombin generation assay was conducted as previously described with modifications [30]. Washed platelets from WT and ERp18-KO mice (1 × 10^8^/mL) were suspended in Tyrode’s buffer. The washed platelets were stimulated with thrombin and stirred (800 rpm/min) for 8 minutes. Exposed phosphatidylserine was detected using FITC-annexin V. Samples were diluted 1:5 for staining with annexin V conjugate in an annexin binding buffer for 15 minutes in the dark. The samples were promptly analyzed using flow cytometry.

### 2.21 Detection of ERp18 in platelet releasate

Washed human platelets (1 × 10^9^/mL) in Tyrode’s buffer were activated with 1 U/mL thrombin for 10 minutes at room temperature with stirring. Cell-free supernatant containing platelet-released proteins was obtained by centrifugation at 16,000 × *g* for 1 minute at room temperature. Soluble platelet-released proteins were enriched by means of a 10 kDa ultra centrifugal filter (UFC5010BK, Millipore) and centrifugation at 14,000 × *g* at 4 °C for 15 minutes. Concentrated platelet-releasate was boiled in 4× SDS−Laemmli buffer with β-mercaptoethanol for 10 minutes at 100 °C and electrophoresed using 10% polyacrylamide gel electrophoresis [16].

### 2.22 Immunofluorescence staining of platelets

After platelet spreading on a coverslip, they were fixed with 4% PFA. Platelets were stained with an anti-ERp18 antibody, followed by incubation with goat anti-rabbit IgG (ab150077, Abcam) and tetramethylrhodamine-conjugated phalloidin. The platelets were imaged using a laser confocal microscope (Olympus).

### 2.23 Statistical analysis

Data analysis was conducted using the statistical software GraphPad Prism 9. For parametric comparisons, values were presented as the mean ± SEM. A 2-tailed Student’s *t-*test for 2 groups and 1-way analysis of variance (anova) followed by Tukey’s test for multiple groups were used. For nonparametric comparisons of the area under the curve in the laser-injury experiments, the Wilcoxon–Mann–Whitney U-test was used. A *P* value of less than .05 was considered significant.

## 3 Results

### 3.1 Generation and characterization of ERp18-KO mice

**Figure 1:**
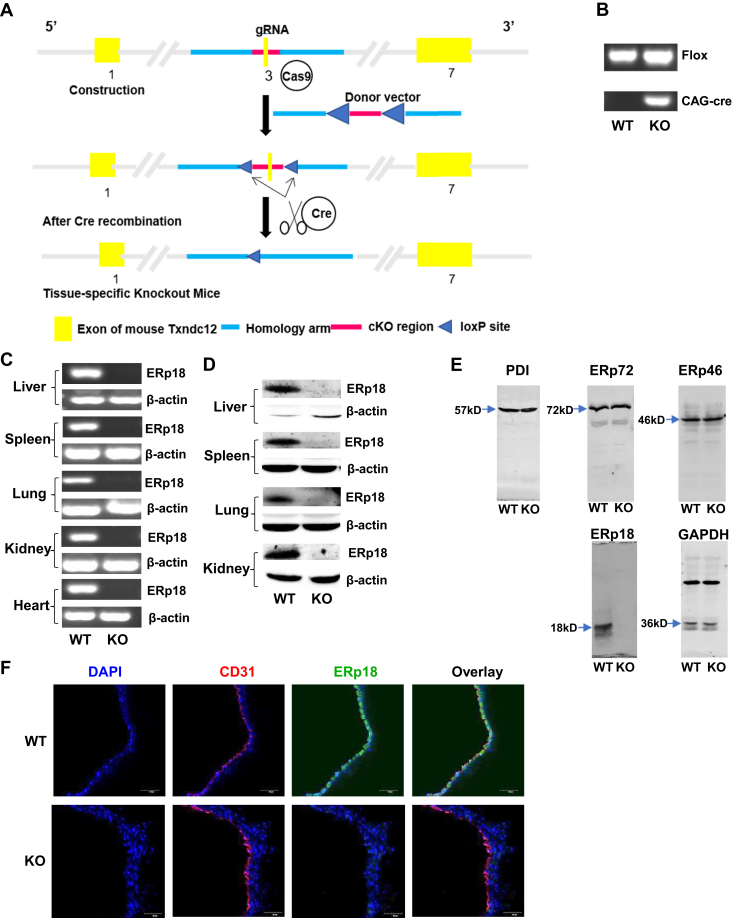
Generation and characterization of ERp18 knockout (KO) mice. (A) A schematic diagram of mouse ERp18 gene deletion. ERp18-floxed mice were generated by clustered regularly interspaced short palindromic repeats/Cas9 technology, and ERp18-KO mice were obtained by mating ERp18-floxed mice with CAG-Cre mice. (B) Genotyping of wild-type (WT; ERp18^flox/flox^) mice and ERp18-KO (CAG-Cre/ERp18^flox/flox^) mice using tail DNA. The bands represent polymerase chain reaction products of the floxed allele (upper panel, 344 bp) and the CAG-Cre gene (lower panel, 337 bp). (C) ERp18 mRNA expression in different organs from WT and KO mice was measured by reverse transcription-polymerase chain reaction with β-actin as a loading control. (D) ERp18 protein expression in different organs from WT and KO mice was detected by Western blotting, with β-actin as the loading control. (E) Expression of ERp18, protein diisulfide isomerase (PDI), ERp72, and ERp46 in platelets from WT and KO mice was evaluated by immunoblotting, with GAPDH as a loading control. (F) Absence of ERp18 expression (green) in the mouse aorta between WT and KO mice was indicated by anti-ERp18 labeling in confocal fluorescence microscopy images; anti-CD31 (red) and 4′,6-diamidino-2-phenylindole (DAPI) (blue) were used as markers of endothelial cells and nuclei. The scale bar was 50 μm. cKO, conditional knockout; gRNA, guide RNA.

**Figure 2:**
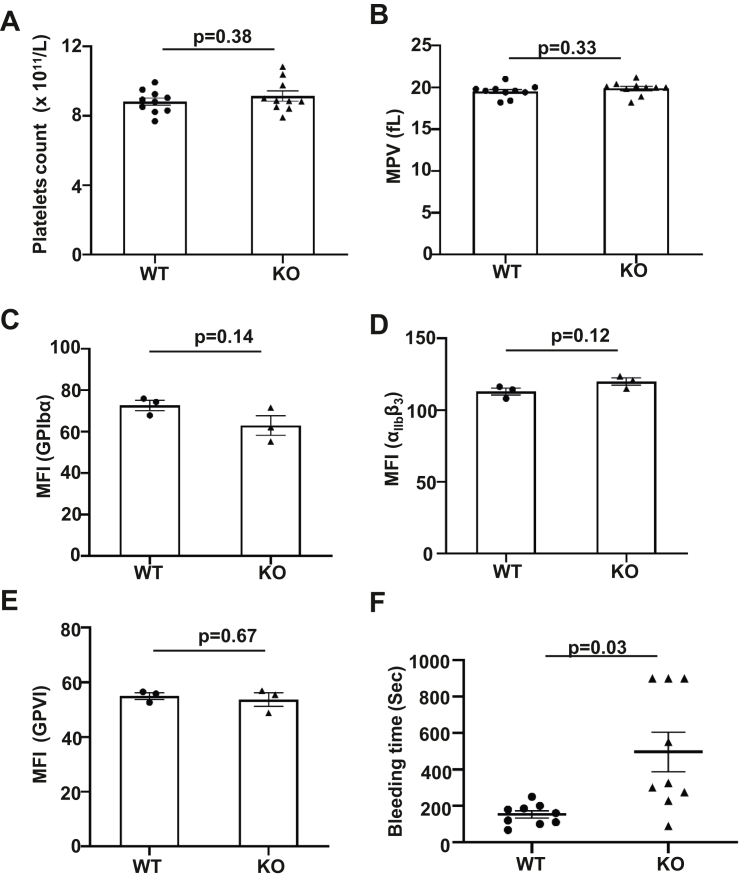
Knockout (KO) mice have comparable platelet counts and major platelet glycoproteins (GPs) expression to wild-type (WT) mice but have prolonged tail bleeding times. (A and B) Comparison of platelet counts and mean platelet volume (MPV) between WT mice and KO mice. Mean ± SEM, ns, not significant, *n* = 10, *t*-test. (C–E) Expression of GPs (α_IIb_β_3_, GPIbα, and GPVI) on platelets from WT mice and KO mice were analyzed by flow cytometry. Mean fluorescent intensity (MFI) is shown in the right panels; mean ± SEM, ns, not significant, *n* = 3, *t*-test. (F) Comparison of tail bleeding time between WT and KO mice. Mean ± SEM, ∗*P* < .05, *n* = 9, *t*-test.

To determine the role of ERp18 in platelet function and arterial thrombus formation, we generated whole-body ERp18-KO mice by introducing loxP sites flanking exon 3 of the ERp18 gene combined with CAG-Cre to induce a KO first strain (Figure 1A). CAG-Cre/ERp18^fl/fl^ (KO) mice showed a homozygous floxed allele (344 bp) and the presence of the CAG-Cre gene (377 bp), in contrast to ERp18^fl/fl^ littermates (WT) without the CAG-Cre gene (Figure 1B). In major organs, including the liver, spleen, kidney, lung, and heart, ERp18-KO mice did not express ERp18 mRNA and protein (Figure 1C, D). In ERp18-KO platelets, ERp18 protein was absent, whereas other thiol isomerases PDI, ERp72, and ERp46 remained comparable to WT platelets, indicating that the loss of ERp18 does not change the expression of other enzymes (Figure 1E). Confocal fluorescence microscopy detected ERp18 expression in endothelial cells in arterial sections of WT mice but not in ERp18-KO mice (Figure 1F). Together, these data indicate successful ERp18 gene deletion. Additionally, ERp18-KO mice had normal platelet counts and platelet size (Figure 2A, B) and a comparable expression of platelet receptors GPIbα, α_IIb_β_3_, and GPVI with WT mice (Figure 2C–E), suggesting that ERp18 is not involved in platelet production and GP synthesis.

### 3.2 ERp18 is required for hemostasis, platelet accumulation, and fibrin formation

**Figure 3:**
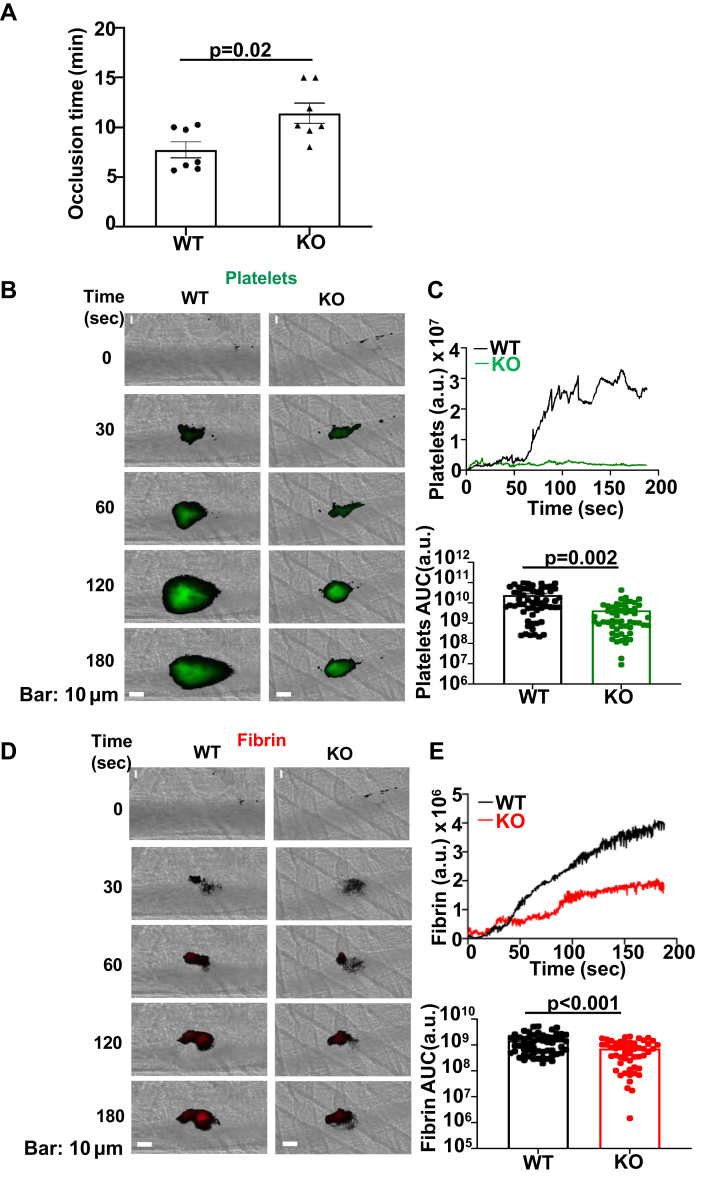
ERp18 deficiency inhibits platelet accumulation and fibrin formation in arterial thrombosis. (A) Time to occlusion of FeCl_3_-induced carotid artery injury in wild-type (WT) and knockout (KO) mice. Mean ± SEM, ∗*P* < .05, *n* = 5, *t*-test. (B–E) Cremaster arteriole injury was induced in WT and KO mice. Platelets and fibrin accumulated at the site of injury were detected using anti-CD41 F(ab)2 fragments conjugated to Alexa Fluor 488 (green) and antifibrin antibody conjugated to Alexa Fluor 647 (red). (B and D) Representative images of (B) platelets and (D) fibrin in the cremaster arterioles at different time points following vessel injury in WT and KO mice are shown. Scale bar, 1 μm. (C and E) The median fluorescence intensity of (C) platelets and (E) fibrin from each group are plotted over time. The area under the curve (AUC) for platelets and fibrin from each individual thrombus in different groups is presented and analyzed for statistical significance. Mean ± SEM, ∗*P* < .05, ns, not significant, *n* ≥32 from 3 mice, *t*-test. a.u., arbitrary unit.

To determine the role of ERp18 in hemostasis, the tail bleeding time was assessed. ERp18-KO mice had a significantly prolonged bleeding time compared with WT mice (Figure 2F), suggesting that ERp18 is required for hemostasis. We next evaluated the function of ERp18 in arterial thrombus formation. In FeCl_3_-induced carotid injury, ERp18-KO mice had a longer time to complete occlusion following FeCl_3_ injury to the carotid artery (Figure 3A). Next, we confirmed the role of ERp18 in platelet accumulation in a laser-induced injury model [31,32]. In cremaster arterial injury induced by laser, ERp18 deficiency significantly inhibited platelet accumulation and fibrin deposition (Figure 3B–E). Because both platelets and endothelial cells express ERp18, the role of platelet ERp18 in thrombosis was assessed using the replacement of platelets in ERp18-KO mice with WT mouse platelets in a FeCl_3_-induced mesenteric thrombosis model. The incorporation of platelets into thrombi in ERp18-KO mice was significantly reduced compared with WT mice, consistent with the results observed in the cremaster arterial injury model. In ERp18-KO mice that were depleted of platelets and infused with WT mouse platelets, their platelet accumulation was higher than that in ERp18-KO mice but lower than that in WT mice (Supplementary Figure S7A, B). These data suggest that both platelet and endothelial ERp18 contribute to arterial thrombosis.

### 3.3 ERp18 deficiency impairs platelet function

**Figure 4:**
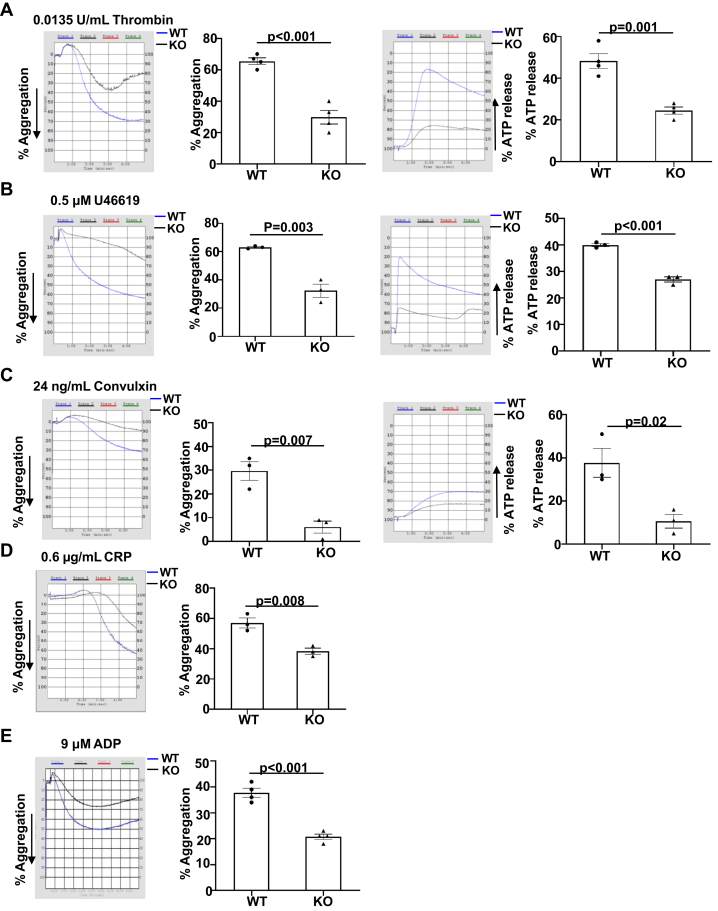
ERp18 deficiency inhibits platelet aggregation and adenosine triphosphate (ATP) release. (A–E) Platelets isolated from wild-type (WT) and knockout (KO) mice were treated with (A) thrombin, (B) U46619, (C) convulxin, (D) collagen-related peptide (CRP), or (E) adenosine diphosphate (ADP). (A–E) Representative aggregation, (A–C) ATP release tracings, and combined results with statistical analysis are shown. Mean ± SEM, ∗*P* < .05, ∗∗*P* < .01, ∗∗∗*P* < .001, *n* = 3 or 4, *t*-test.

**Figure 5:**
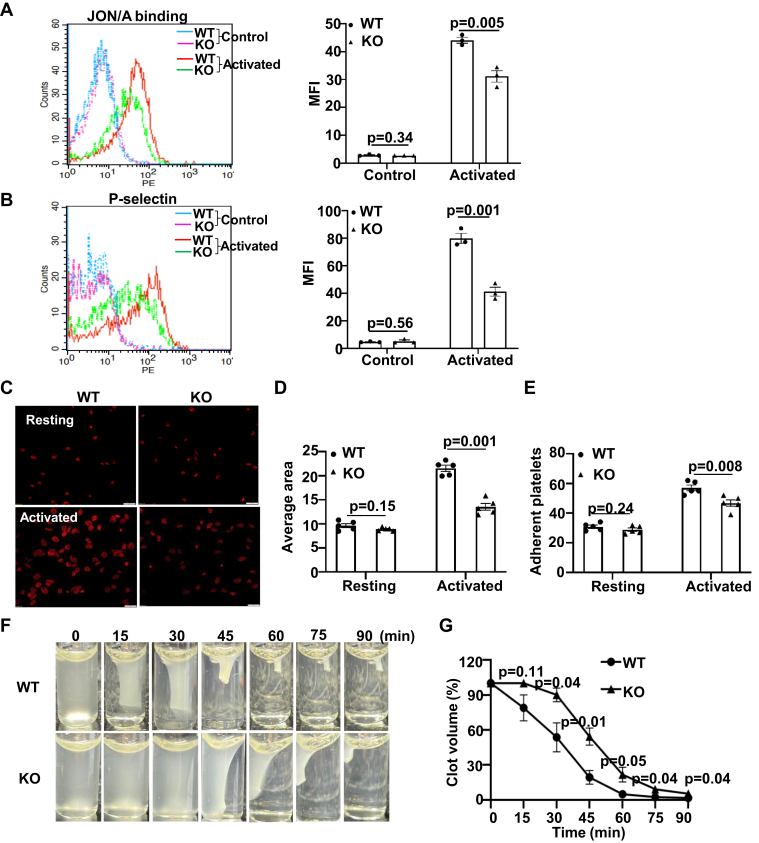
ERp18-deficient platelets have a decrease in integrin α_IIb_β_3_ activation, adhesion, and clot retraction. (A and B) ERp18-deficient platelets had defective thrombin (0.012 U/mL)-induced (A) integrin α_IIb_β_3_ activation (JON/A antibody binding) and (B) P-selectin expression. A representative histogram (left) and combined results (right) are presented. Mean fluorescent intensity (MFI) ± SEM, ∗∗*P* < .01, ns, not significant, *n* = 3, *t*-test. (C–E) Washed platelets or platelets treated with 0.014 U/mL thrombin were placed on glass coverslips precoated with 5 μg/mL fibrinogen at 37 °C for 40 minutes. Adherent cells were permeabilized and stained with tetramethylrhodamine-phalloidin, and adhesion was evaluated using ImageJ software. (C) Representative images. Scale bar, 10 μm. (D) The number of platelets adhered to fibrinogen-coated glass coverslips and (E) quantification of the average surface area of individual platelets are shown. Mean ± SEM, ∗∗*P* < .01, ns, not significant, *n* = 5, *t*-test. (F and G) Clot retraction was initiated with 0.1 U/mL thrombin, and images were captured at the specified times. (F) Representative clot retraction images. (G) Two-dimensional clot size was measured using ImageJ and normalized to the clot size at time 0 (%). Mean ± SEM, ∗*P* < .05, *n* = 4, *t*-test. KO, knockout; WT, wild-type.

The defect in hemostasis and thrombosis of ERp18-KO mice suggests that ERp18 is important for platelet function. In response to different receptor agonists, including thrombin, convulxin, and U46619, ERp18-deficient platelets displayed a significant decrease in aggregation and ATP release compared with WT platelets (Figure 4A–C). Moreover, aggregation of ERp18-deficient platelets induced by adenosine diphosphate or collagen-related peptide was also decreased (Figure 4D, E). However, in response to higher concentrations of these agonists, aggregation and ATP release of ERp18-deficient platelets were comparable to WT platelets (Supplementary Figure S1), suggesting the possibility that high concentrations of the agonists stimulate abundant other thiol isomerases, which compensate for the absence of ERp18. As detected by flow cytometry, ERp18-deficient platelets showed a significant decrease in integrin α_IIb_β_3_ activation (JON/A) and P-selectin expression induced in response to 0.012 U/mL thrombin (Figure 5A, B), whereas a higher concentration of thrombin (0.024 U/mL) overcame the defective integrin α_IIb_β_3_ activation and P-selectin expression (Supplementary Figure S2A, B). Platelet spreading and clot retraction were further used to verify the role of ERp18 in integrin α_IIb_β_3_ outside-in signaling [33]. ERp18 deficiency decreased the number and area of adherent platelets on a fibrinogen-coated surface compared with WT platelets (Figure 5C–E). Besides, clot retraction of ERp18-KO platelets was significantly delayed (Figure 5F, G). It is possible that ERp18 deficiency inhibits the ligand binding affinity of integrin α_IIb_β_3_, and the defect might be compensated for by other thiol isomerases over time. However, ERp18 deficiency had no influence on Ca^2+^ mobilization (Supplementary Figure S3E, F). Taken together, these data indicate that ERp18 is critical for platelet functions, including aggregation, granule release, adhesion, and clot retraction.

### 3.4 ERp18 binds to integrin α_IIb_β_3_ on platelets and reduces its disulfides

**Figure 6:**
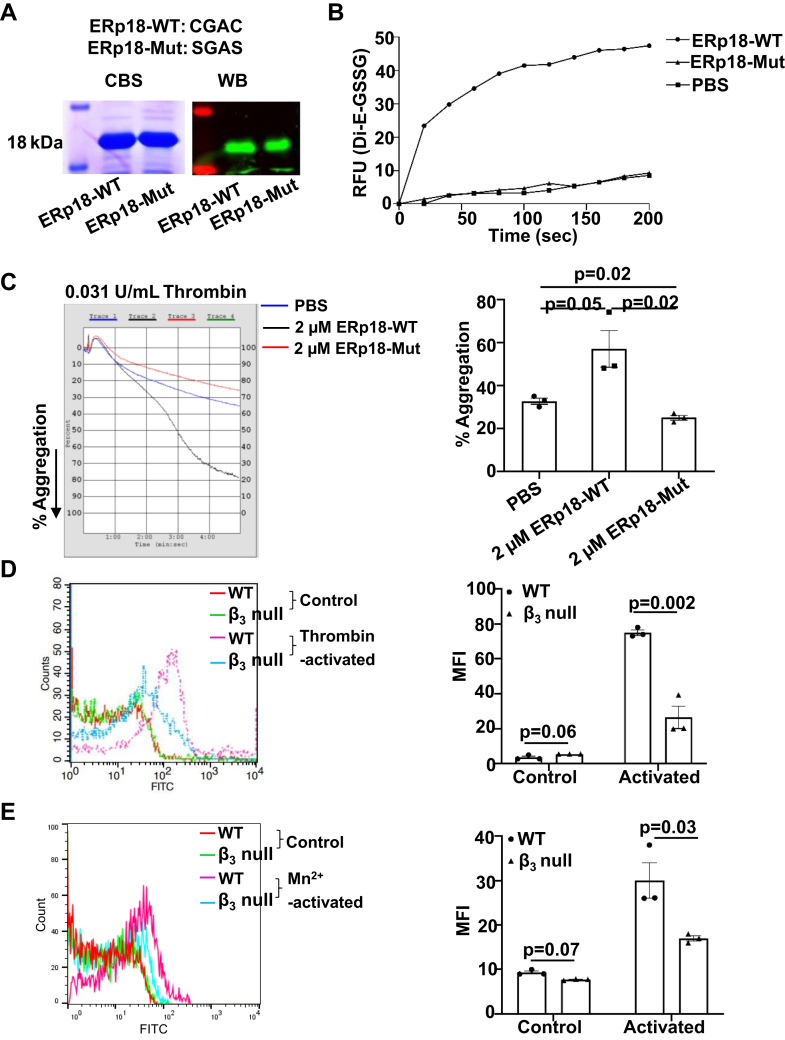
ERp18 enhances platelet aggregation by binding to integrin α_IIb_β_3_. (A) Recombinant ERp18 wild-type (ERp18-WT; CGAC) and ERp18 mutant (ERp18-Mut; SGAS) proteins were verified by Coomassie blue staining (left) and Western blotting (right). (B) A dieosinediglutathione (Di-E-GSSG) substrate cleavage assay was conducted to determine the reductase activity of ERp18-WT and ERp18-Mut proteins. Phosphate-buffered saline (PBS) was used instead of ERp18 protein as a negative control. (C) Washed human platelets were pretreated with ERp18-WT or ERp18-Mut and then stimulated with thrombin (0.031 U/mL) to induce platelet aggregation. Mean ± SEM, ∗*P* < .05, *n* = 3, anova. (D and E) ERp18 binding to β_3_ integrin on platelets. Washed platelets from wild-type (WT) and β_3_-null mice (2 × 10^7^/mL) were preincubated with fluorescein isothiocyanate (FITC)-conjugated ERp18-WT protein for 10 minutes at room temperature, followed by stimulation with (D) 0.1 U/mL thrombin or (E) 2 mM Mn^2+^ for 5 minutes. Surface binding of FITC-conjugated ERp18 was detected by flow cytometry. Representative histogram (left) and combined results with statistical analysis (right); mean fluorescent intensity (MFI) ± SEM, ∗∗*P* < .01, ∗∗∗*P* < .001, ns, not significant, *n* = 3, *t*-test.

**Figure 7:**
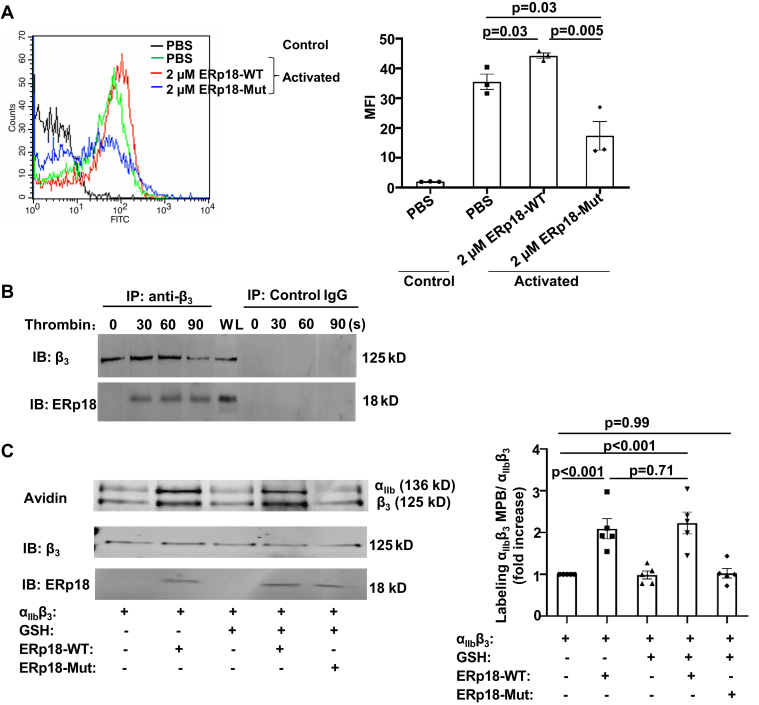
ERp18 is associated with integrin α_IIb_β_3_ and reduces its disulfide. (A) Washed human platelets (2 × 10^7^/mL) were pretreated with 2 μM ERp18 wild-type (ERp18-WT) or ERp18 mutation (ERp18-Mut) protein at 37 °C, followed by activation with 0.1 U/mL thrombin in the presence of fluorescein isothiocyanate (FITC)-conjugated fibrinogen. The binding of fibrinogen was measured by flow cytometry. A representative histogram (left) and combined mean fluorescent intensity (MFI) results (right) are shown. Mean ± SEM, ∗*P* < .05, ∗∗*P* < .01, *n* = 3, anova. (B) Washed human platelets (1 × 10^9^/mL) were stimulated with thrombin (0.5 U/mL) for the indicated times. After lysis, integrin α_IIb_β_3_ was immunoprecipitated with anti-β_3_ antibody SZ-21 and protein G agarose. Identical samples were also incubated with control immunoglobulin (Ig) G. Immunoblotting was performed with a goat anti-ERp18 antibody and monoclonal anti-mouse anti-β_3_ antibody. (C) Purified human α_IIb_β_3_ protein was incubated with (+) or without (−) 2 μM ERp18 protein and glutathione (GSH), followed by labeling with 3-(N-maleimido-propionyl) biocytin (MPB). MPB-labeled α_IIb_ and β_3_ were detected by blotting with avidin-horseradish peroxidase. Immunoblotting with anti-β_3_ and anti-ERp18 antibodies indicates equal protein loading. The left panel is a representative blot; the right panel is a quantitative analysis of MPB labeling level by densitometry of band density. Mean ± SEM, ∗∗*P* < .01, ns, not significant, *n* = 5, 1-way anova. IB, immunoblotting; IP, immunoprecipitation; WL, whole cell lysate.

To understand the role of ERp18 in platelet function and its substrates, we examined the interaction between ERp18 and integrin α_IIb_β_3_. ERp18-WT (CGAC) protein and inactive ERp18-Mut (SGAS) protein, which lost the catalytic function, were generated (Figure 6A). In a reductase assay using Di-E-GSSG, ERp18-WT protein, but not ERp18-Mut protein, exhibited reductase activity (Figure 6B). ERp18-Mut, which has lost its catalytic activity, still retains the ability to bind to substrates. When added to platelets, the ERp18-Mut protein could compete with the secreted ERp18 from platelets for substrate binding, thereby diminishing the catalytic effect of ERp18. In the platelet aggregation assay, compared with the control, ERp18-WT protein enhanced human platelet aggregation induced by thrombin, whereas ERp18-Mut protein had an inhibitory effect (Figure 6C). The effects of these proteins were dose-dependent (Supplementary Figure S5B, C). These data suggest that the enzymatic activity of ERp18 is necessary for the regulation of human platelet aggregation. Similar to the effect on human platelets, the addition of ERp18-WT protein to ERp18-KO platelets increased aggregation to a comparable level of WT platelets (Supplementary Figure S5A). To understand whether ERp18 interacts with integrin α_IIb_β_3_, we first examined the binding of ERp18 to integrin α_IIb_β_3_ on the activated platelet surface. As detected by flow cytometry, ERp18-WT protein bound to WT mouse platelets after activation with thrombin or Mn^2+^. However, the binding of the ERp18-WT protein to β_3_-null platelets was markedly decreased (Figure 6D, E), suggesting that ERp18 interacts with the platelet surface primarily through integrin α_IIb_β_3_. Additionally, ERp18 may also bind to platelets via an α_IIb_β_3_-independent mechanism, potentially involving integrin α_2_β_1_ and GPIbα. Next, the impact of ERp18 on the ligand binding affinity of integrin α_IIb_β_3_ was evaluated by measurement of fibrinogen binding to platelets. ERp18-WT facilitated the binding of fibrinogen to thrombin-activated human platelets, while the inactive ERp18-Mut inhibited the binding (Figure 7A), suggesting that ERp18 enhances the ligand binding affinity of integrin α_IIb_β_3_. The addition of ERp18-WT protein to ERp18-KO platelets also enhanced the binding of fibrinogen (Supplementary Figure S6A), consistent with its effect on ERp18-KO platelet aggregation (Supplementary Figure S5A). Meanwhile, the ERp18-Mut protein inhibited the binding of activated platelets to fibrinogen in a dose-dependent manner (Supplementary Figure S6B). Additionally, we detected an interaction between ERp18 and integrin α_IIb_β_3_ in activated platelets using a coimmunoprecipitation method (Figure 7B). Disulfide breakage of integrin α_IIb_β_3_ contributes to its conformational changes to form a high ligand binding affinity that is needed for platelet aggregation [34,35]. Given that ERp18 had reductase activity (Figure 6B), we proceeded to examine whether ERp18 protein affects the thiol-disulfide exchange of integrin α_IIb_β_3_ using the MPB labeling technique. When ERp18-WT protein was incubated with purified integrin α_IIb_β_3_ protein, it, but not ERp18-Mut, enhanced the thiol labeling of integrin α_IIb_β_3_ (Figure 7C), indicating that ERp18 cleaves the disulfide bonds of integrin α_IIb_β_3_, leading to subsequent structural changes for high-affinity ligand binding.

## 4 Discussion

In this study, using a new genetically modified mouse model deficient in ERp18, we provide the first evidence demonstrating that ERp18 plays an important role in hemostasis, thrombosis, and platelet function. Previously, we and others have identified 7 thiol isomerases that regulate arterial thrombosis and platelet function, including PDI, ERp46, ERp57, ERp72, ERp5, TMX1, and TMX4 [10]. All of these enzymes contain a CXXC active site, indicating a critical role for thiol isomerases with the CXXC active site in the regulation of thrombosis. Our new findings show the role of ERp18, which also contains a CXXC active site, highlighting the complexity of the redox network formed by CXXC-containing thiol isomerases in the regulation of platelet function and thrombosis. In this study, using the ERp18 antibody, ERp18 was not detected in either mouse or human plasma at the resting states, suggesting that ERp18 is not secreted from resting platelets (Supplementary Figure S4A). ERp18 was found on the surface of activated platelets and released in the supernatant (Supplementary Figure S4B–D). Utilizing the newly generated ERp18-KO mice, we found that the deficiency of ERp18 significantly prolonged tail bleeding time (Figure 2). In FeCl_3_-mediated carotid and mesenteric artery injury and laser-induced cremaster arterial injury models, ERp18-KO mice exhibited a significant decrease in thrombus formation (Figure 3 and Supplementary Figure S7). Since the deficiency of platelet ERp18 did not reduce thrombin generation (Supplementary Figure S3A–C) and phosphatidylserine exposure (Supplementary Figure S3D), it could be concluded that ERp18 does not contribute to platelet procoagulant activity. The diminished fibrin formation observed in ERp18-KO mice in the laser-induced injury model is likely the result of decreased platelet accumulation. Collectively, these data demonstrate that ERp18, like other PDIs, is secreted from activated platelets and plays a critical biological role of ERp18 in facilitating hemostasis and thrombosis.

Platelets play a crucial role in hemostasis and thrombosis. In response to agonists of various receptors, including thrombin, collagen, thromboxane A2, and adenosine diphosphate, ERp18-deficient platelets exhibited a decrease in platelet aggregation and ATP release (Figures 4 and 5), indicating that ERp18 contributes to the general mechanism of platelet activation. Furthermore, ERp18 deficiency inhibited P-selectin expression, integrin α_IIb_β_3_ activation, adhesion, and clot retraction in platelets (Figures 5 and 6), supporting that ERp18 is essential for platelet function. Upon platelet activation, the activation of primary receptors triggers inside-out signaling, resulting in conformational changes of integrin α_IIb_β_3_ that enhance its ligand binding affinity. Consequently, ligation-induced outside-in signaling promotes platelet spreading and clot retraction. During the affinity transition of integrin α_IIb_β_3_, the cleavage of its disulfide bonds is essential [34]. In this study, we found that ERp18 targets the disulfides of integrin α_IIb_β_3_ as one of its substrates. First, the close interaction between ERp18 and integrin α_IIb_β_3_ is shown by flow cytometry (Figure 6) and coimmunoprecipitation (Figure 7). Consistent with the physical interaction, ERp18 exhibited a strong capacity to reduce the disulfides of integrin α_IIb_β_3_ (Figure 7), which is a potential mechanism by which ERp18 regulates platelet activation. Integrin α_IIb_β_3_ contains numerous disulfide bonds [[36], [37], [38]]; the specific disulfides targeted by ERp18 in α_IIb_ and β_3_ subunits are currently under investigation. Knowledge of the breakage and formation of specific functional disulfides in α_IIb_β_3_ integrin that are catalyzed by thiol isomerases, including ERp18, is crucial for understanding the redox mechanism underlying platelet activation. Structurally, ERp18 features a single catalytic domain with a CGAC active site motif and an insertion between the β_3_ and α_3_ regions of the thioredoxin fold [17]. The specific domains of ERp18 that are responsible for the dynamic binding of integrin α_IIb_β_3_ are currently under investigation.

Our findings regarding the role of ERp18 in thrombosis once again raise the question: why are so many thiol isomerases needed in this process? While there may be some distinction between oxidation and reductase functions, there is clear specificity in the types of client proteins that each enzyme interacts with. Based on the previous studies from other laboratories and our own, the deletion of any one thiol isomerase—whether it be ERp57, ERp72, ERp46, PDI, TMX4, or now ERp18—did not affect the expression of the others. However, it did impact platelet responsiveness and thrombosis, suggesting that each of these enzymes is essential for these processes. ERp18 exhibited functions in platelets that are similar to those of other isomerases, including platelet aggregation and granule release. However, unlike ERp72 and ERp5, ERp18 does not participate in Ca^2+^ mobilization, which is similar to PDI [3,39]. The functions of various hemostatic proteins in platelet activation (such as integrin α_IIb_β_3_, integrin α_2_β_1_, GPIbα, and GPVI), as well as coagulation factors (like tissue factor and factor XI) and plasma proteins (including von Willebrand factor, fibrinogen, and vitronectin), are all regulated by thiol-disulfide exchanges [40]. Furthermore, the redox reactions involving multiple cysteine residues in each protein play a significant role in thrombosis, particularly in the integrin β_3_ subunit. It is unlikely that a single thiol isomerase could fulfill the redox reactions involved in the initiation and maturation of thrombosis; therefore, their cooperation is essential [41]. It is possible that ERp18 regulates the cysteines in these hemostatic proteins that are not targeted by other isomerases, and PDI, ERp46, ERp57, and ERp72 may not compensate for the absence of ERp18 in platelets. Currently, we are employing differential iodo-tandem mass tag labeling and mass spectrometry to compare the ratio of functional cysteines in major platelet proteins between ERp18-KO platelets and other thiol isomerase-deficient platelets. This approach will enhance our understanding of the specific substrates and the targeted cysteines of ERp18 in platelets. This study is focused on the role of ERp18 in the function of platelets, which is a crucial component of arterial thrombosis. Since endothelial cells also play a pivotal role in this process, we plan to address the role of endothelial ERP18 utilizing an endothelial cell-specific KO model, such as vascular endothelial cadherin-Cre/ERp18^fl/fl^ mice, in our future research.

Our findings in the current study demonstrate that ERp18 is a novel thiol isomerase that enhances platelet activation and thrombosis. We have identified 7 thiol isomerases that promote arterial thrombosis and platelet function: PDI, ERp46, ERp57, ERp72, ERp5, TMX4, and ERp18, all of which contain a CXXC active site. The newly discovered function of ERp18 highlights the complexity of the redox network formed by these positive thiol isomerases in regulating platelet function and thrombosis. Further understanding of the mechanisms underlying the specific role of ERp18 and other thiol isomerases is crucial, as it will not only provide new insights into the platelet activation process and the pathogenesis of thrombotic diseases but also identify which enzymes may serve as the most promising antithrombotic targets.

## Supplementary material

Supplemental Figure S1 Higher doses of thrombin, U46619, convulxin, ADP, or CRP overcome defective platelet aggregation and ATP secretion of ERp18-deficient platelets. (A–E) Platelets isolated from WT and KO mice were treated with (A) thrombin, (B) U46619, (C) convulxin, (D) CRP, or (E) ADP. Representative aggregation and ATP release tracings and combined results with statistical analysis were shown. The defects were overcome with higher doses of (A) thrombin (0.03 U/mL), (B) U46619 (1 μM), (C) convulxin (48 ng/mL), (D) ADP (18 μM), or (E) CRP (1.2 μg/mL). Mean ± SEM, ns, not significant, *n* = 3, *t*-test. **Supplemental Figure S2** Higher concentrations of Thrombin (0.024 U/mL) overcome defective activation of α_IIb_β_3_ and P-selectin expression of ERp18 deficient platelets. (A-B) ERp18-deficient platelets had defective thrombin (0.024 U/mL)- induced integrin α_IIb_β_3_ activation (JON/A antibody binding) and P-selectin expression. Representative histogram (left) and combined results (right). MFI ± SEM, ns, not significant, *n* = 3, *t*-test. **Supplemental Figure S3** The effects of ERp18 on platelet procoagulant activity. (A–F) The effects of ERp18 on platelet procoagulant activity were determined using a thrombin generation assay. PRP, PPP, or plasma was obtained from WT and KO mice. Thrombin generation in PRP and plasma was triggered by 1.0 pM tissue factor (A and D) and 5 pM tissue factor plus 4 μM phospholipids (C and F), respectively. Referring to the thrombin generation in plasma supplemented with washed platelets, thrombin (0.1 U/mL) plus collagen (10 μg/mL) stimulated washed WT or KO platelets were used to initiate the reaction (B and E). Representative traces (upper panel) and peak thrombin generation (lower panel) were shown. Mean ± SEM, ns, not significant, *n* = 3, *t*-test. G. Thrombin-induced (0.05 U/mL) phosphatidylserine exposure in washed platelets from WT or KO mice was examined using FITC-conjugated annexin V on flow cytometry. A typical flow cytometry histogram and the percentage of phosphatidylserine positive population were analyzed. MFI ± SEM, ns, not significant, *n* = 3, *t*-test. (H and I) Thrombin-induced (0.08U/mL) Ca^2+^ mobilization in platelets, as determined by Fluo-4 fluorescent signal, was monitored over time (H) by flow cytometry, MFI ± SEM, ns, not significant, *n* = 3 and (I) microplate reader. Mean RFU ± SEM, ns, not significant, *n* = 3 to 7. RFU, relative fluorescence units. **Supplemental Figure S4** Detection of ERp18 expression and secretion. (A) ERp18 was not detected in human and mouse platelet-poor plasma using western blotting. (B-C) Expression of ERp18 on the surface of nonactivated platelets and Thrombin (1 U/mL)-activated platelets. Normal rabbit IgG (nI IgG, 50 μg/mL) or anti-ERp18 antibody (50 μg/mL) was added, and goat anti-rabbit (Alexa Fluor 488, ab150077, Abcam) was used as a secondary antibody to detect. MFI ± SEM, ∗∗*P* < .01, *n* = 3, *t*-test. (D) Thrombin (1 U/mL)-induced platelet activation/aggregation released ERp18 into the supernatant. ERp18 secreted to the supernatant was analyzed by immunoblotting. **Supplemental Figure S5** Role of ERp18 protein in the aggregation of human platelets or KO mouse platelets. (A) Platelets isolated from KO mice were treated with ERp18 protein and 0.014 U/mL Thrombin. Representative aggregation tracings and combined results with statistical analysis were shown. (B and C) Healthy human platelets were treated with ERp18 protein and thrombin. Representative aggregation tracings and combined results with statistical analysis were shown. The ERp18 protein was incubated with the human or ERp18-KO mouse platelets for 5 minutes before detection. Mean ± SEM, ∗*P* < .05, ∗∗*P* < .01, ∗∗∗*P* < .001, ns, not significant, *n* = 3, 1-way anova. **Supplemental Figure S6** The role of ERp18 protein in the binding between platelet and fibrinogen. (A) Washed ERp18 KO mice platelets (2 × 10^7^/mL) were pretreated with 2 μM ERp18-WT protein at 37 °C, followed by activation with 0.01 U/mL thrombin in the presence of FITC-conjugated fibrinogen. (B) Washed human platelets were pretreated with PBS or 1 or 2 μM ERp18-Mut protein at 37 °C, followed by activation with 0.1 U/mL thrombin in the presence of FITC-conjugated fibrinogen. The binding of fibrinogen was measured by flow cytometry. A representative histogram (left) and combined MFI results (right) are shown. MFI ± SEM, ∗*P* < .05, ∗∗*P* < .01, ∗∗∗*P* < .001, *n* = 3, 1-way anova. **Supplemental Figure S7** ERp18 deficiency inhibits platelet accumulation and fibrin formation in arterial thrombosis. (A) Incorporation of platelets into growing thrombus in Group1 (WT), Group2 (KO), and Group3 (KO mice that were depleted of platelets and transfused with WT mouse platelets) was detected using FeCl_3_-induced mesenteric arterial injury, Images at 0, 7, 11, and 15 minutes. The vessel wall was marked by the dotted line. Scale bar, 1 mm. Images were originally magnification X100. (B) Composite of fluorescence intensity (FI) per area analyzed (FI/mm^2^) in 3 groups. Mean ± SEM, ∗*P* < .05, ∗∗*P* < .01, ∗∗∗*P* < .001, *n* = 13 from 5 mice in each group, *t*-test. **Supplemental Figure S8** Detection of ERp18 expression in platelets by immunofluorescence. ERp18 expression (green) in the healthy human platelets indicated by anti-ERp18 labeling in confocal fluorescence microscopy images. F-actin (red) was used as the marker of platelets. The scale bar is 15 μm.
