# Supplementary material for: Thiol isomerase ERp18 enhances platelet activation and arterial thrombosis
Source: Res Pract Thromb Haemost. 2025 Feb 27;9(2):102706. doi: 10.1016/j.rpth.2025.102706 (PMC11986512; doi:10.1016/j.rpth.2025.102706)
Supplement: Supplemental Figure S1 — Higher doses of thrombin, U46619, convulxin, ADP, or CRP overcome defective platelet aggregation and ATP secretion of ERp18-deficient platelets. (A–E) Platelets isolated from WT and KO mice were treated with (A) thrombin, (B) U46619, (C) convulxin, (D) CRP, or (E) ADP. Representative aggregation and ATP release tracings and combined results with statistical analysis were shown. The defects were overcome with higher doses of (A) thrombin (0.03 U/mL), (B) U46619 (1 μM), (C) convulxin (48 ng/mL), (D) ADP (18 μM), or (E) CRP (1.2 μg/mL). Mean ± SEM, ns, not significant, n = 3, t-test. Supplemental Figure S2 Higher concentrations of Thrombin (0.024 U/mL) overcome defective activation of αIIbβ3 and P-selectin expression of ERp18 deficient platelets. (A-B) ERp18-deficient platelets had defective thrombin (0.024 U/mL)- induced integrin αIIbβ3 activation (JON/A antibody binding) and P-selectin expression. Representative histogram (left) and combined results (right). MFI ± SEM, ns, not significant, n = 3, t-test. Supplemental Figure S3 The effects of ERp18 on platelet procoagulant activity. (A–F) The effects of ERp18 on platelet procoagulant activity were determined using a thrombin generation assay. PRP, PPP, or plasma was obtained from WT and KO mice. Thrombin generation in PRP and plasma was triggered by 1.0 pM tissue factor (A and D) and 5 pM tissue factor plus 4 μM phospholipids (C and F), respectively. Referring to the thrombin generation in plasma supplemented with washed platelets, thrombin (0.1 U/mL) plus collagen (10 μg/mL) stimulated washed WT or KO platelets were used to initiate the reaction (B and E). Representative traces (upper panel) and peak thrombin generation (lower panel) were shown. Mean ± SEM, ns, not significant, n = 3, t-test. G. Thrombin-induced (0.05 U/mL) phosphatidylserine exposure in washed platelets from WT or KO mice was examined using FITC-conjugated annexin V on flow cytometry. A typical flow cytometry histogram and the percentage of phosphat [file mmc1.pptx]

## Slide 1
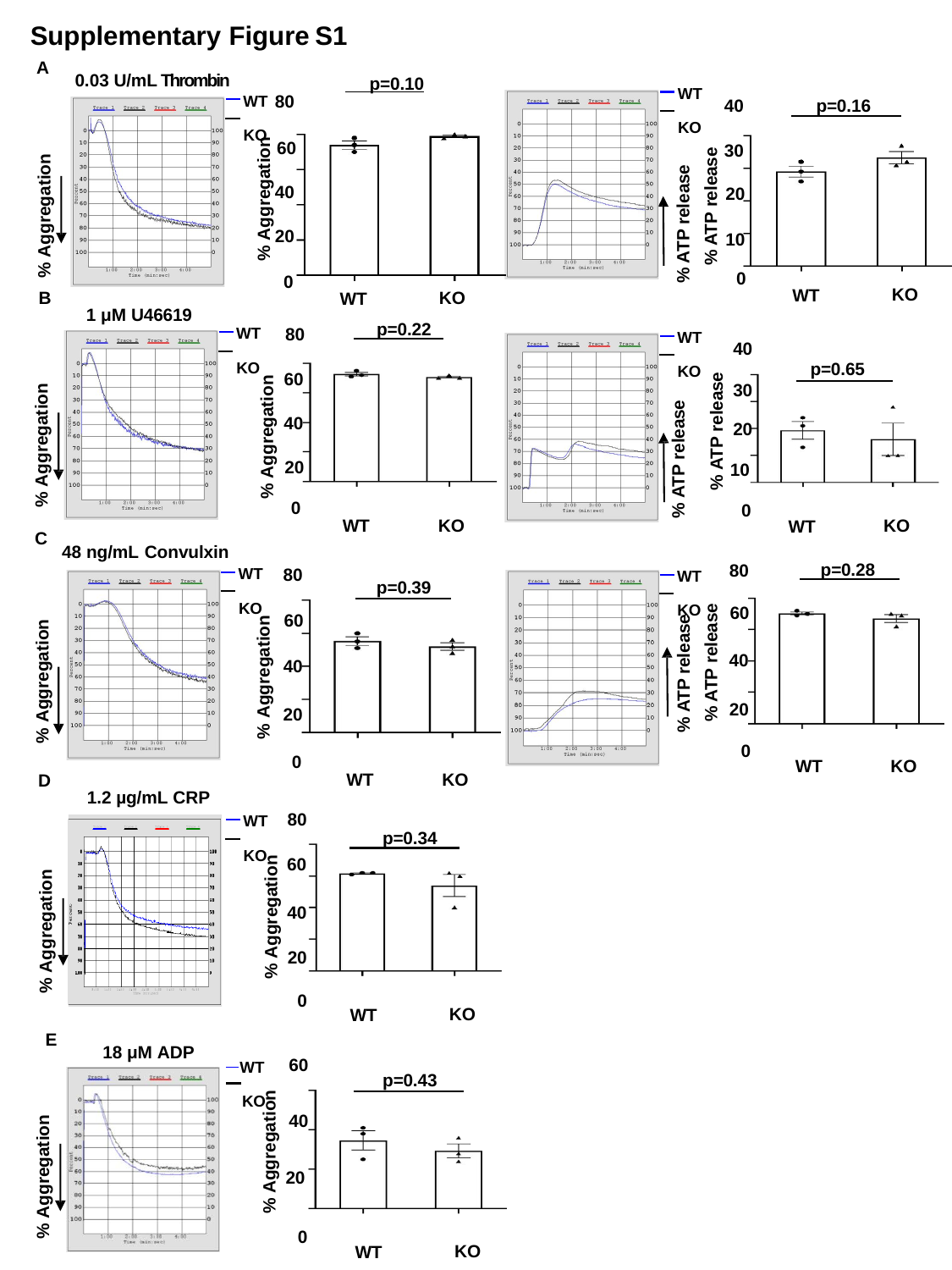

Supplementary Figure S1
A
0.03 U/mL Thrombin
 p=0.10
80
% Aggregation
60
40
20
0
KO
WT
WT KO
WT KO
40
p=0.16
30
% ATP release
20
10
0
KO
WT
% Aggregation
% ATP release
B
1 μM U46619
p=0.22
80
60
% Aggregation
40
20
0
WT
KO
WT KO
WT KO
40
p=0.65
% ATP release
30
20
10
0
KO
WT
% Aggregation
% ATP release
C
48 ng/mL Convulxin
p=0.28
80
% ATP release
60
40
20
0
WT
KO
WT KO
80
p=0.39
60
% Aggregation
40
20
0
WT
KO
WT KO
% ATP release
% Aggregation
D
1.2 µg/mL CRP
80
p=0.34
60
40
20
% Aggregation
0
KO
WT
WT KO
% Aggregation
E
18 μM ADP
60
p=0.43
% Aggregation
40
20
0
KO
WT
 WT KO
% Aggregation

## Slide 2
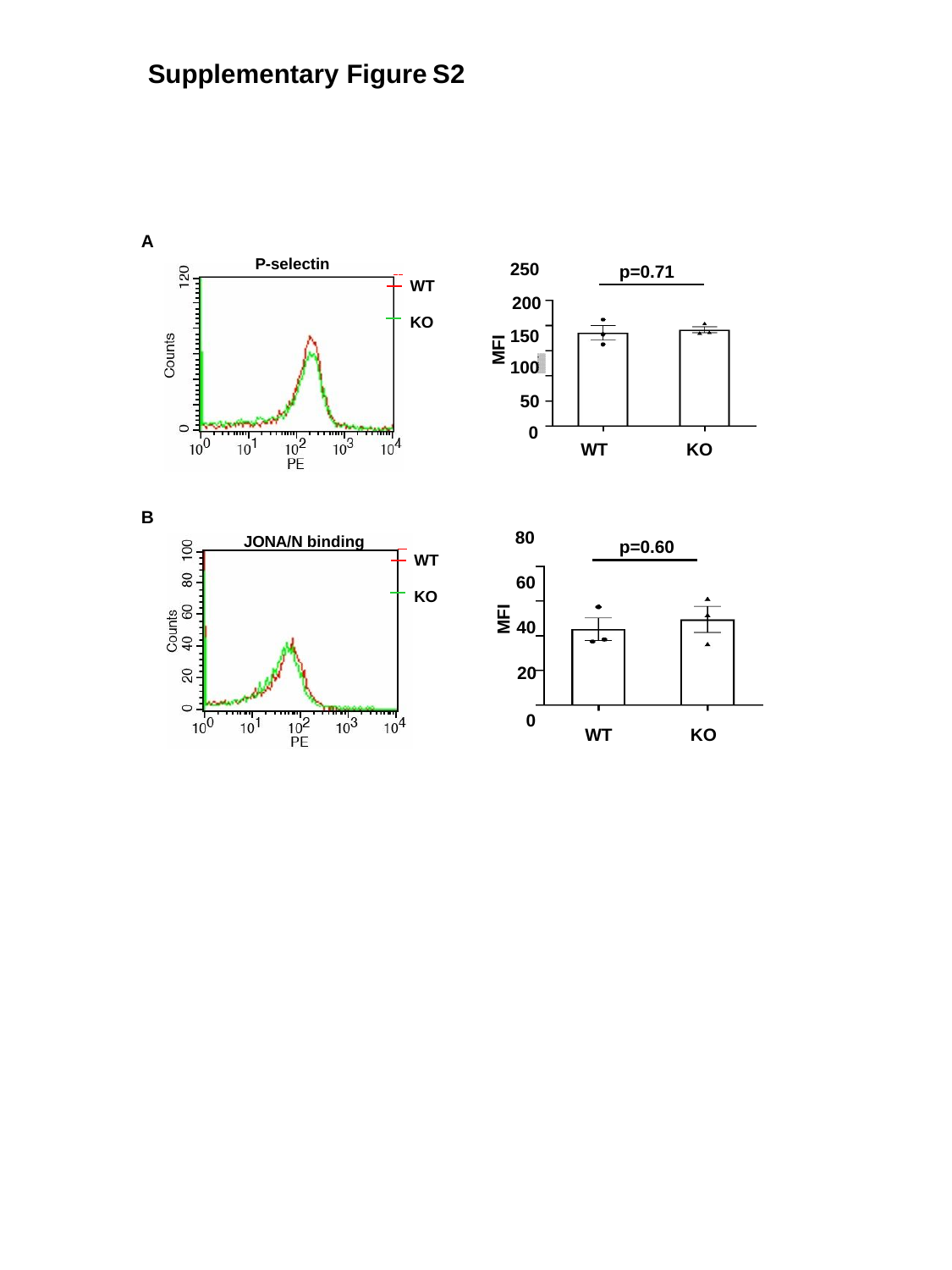

Supplementary Figure S2
A
250
p=0.71
MFI
WT
KO
200
150
100
50
0
P-selectin
WT
KO
B
80
p=0.60
60
MFI
40
20
0
KO
WT
JONA/N binding
WT
KO

## Slide 3
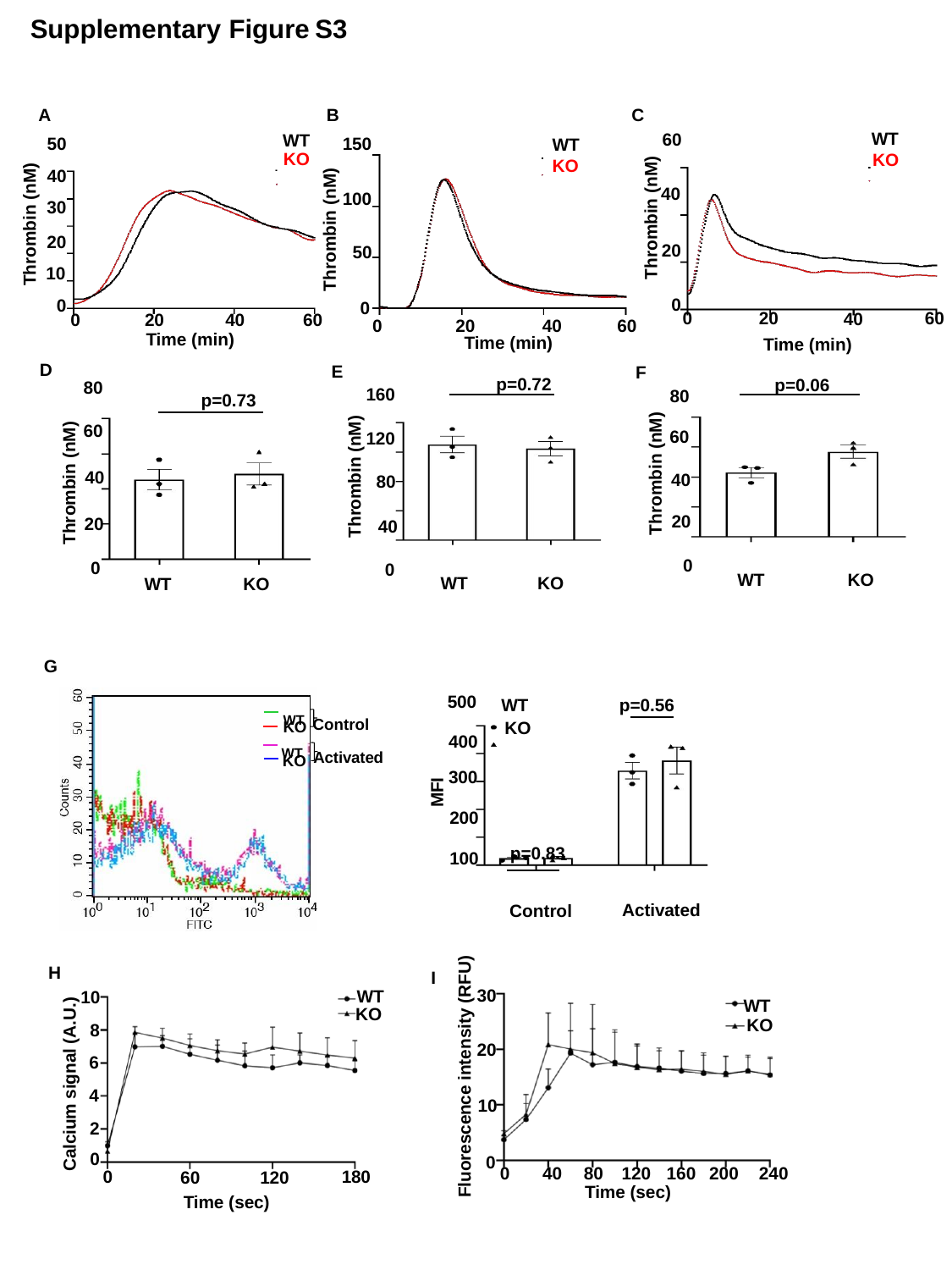

Supplementary Figure S3
C
A
B
WT
60
KO
40
Thrombin (nM)
20
0
0
20
60
40
Time (min)
WT
50
KO
40
30
Thrombin (nM)
20
10
0
0
20
40
60
Time (min)
150
WT
KO
100
Thrombin (nM)
50
0
0
20
40
60
Time (min)
D
E
F
p=0.72
160
Thrombin (nM)
120
80
40
0
WT
KO
p=0.06
80
Thrombin (nM)
60
40
20
0
KO
WT
80
p=0.73
60
Thrombin (nM)
40
20
0
KO
WT
G
p=0.56
500
WT
KO
400
300
MFI
200
p=0.83
100
Activated
Control
WT Control
KO
WT Activated
KO
Fluorescence intensity (RFU)
30
WT
KO
20
10
0
0
40
80
120
160
200
240
Time (sec)
H
I
10
8
6
4
2
WT
Calcium signal (A.U.)
KO
0
0
180
60
120
Time (sec)

## Slide 4
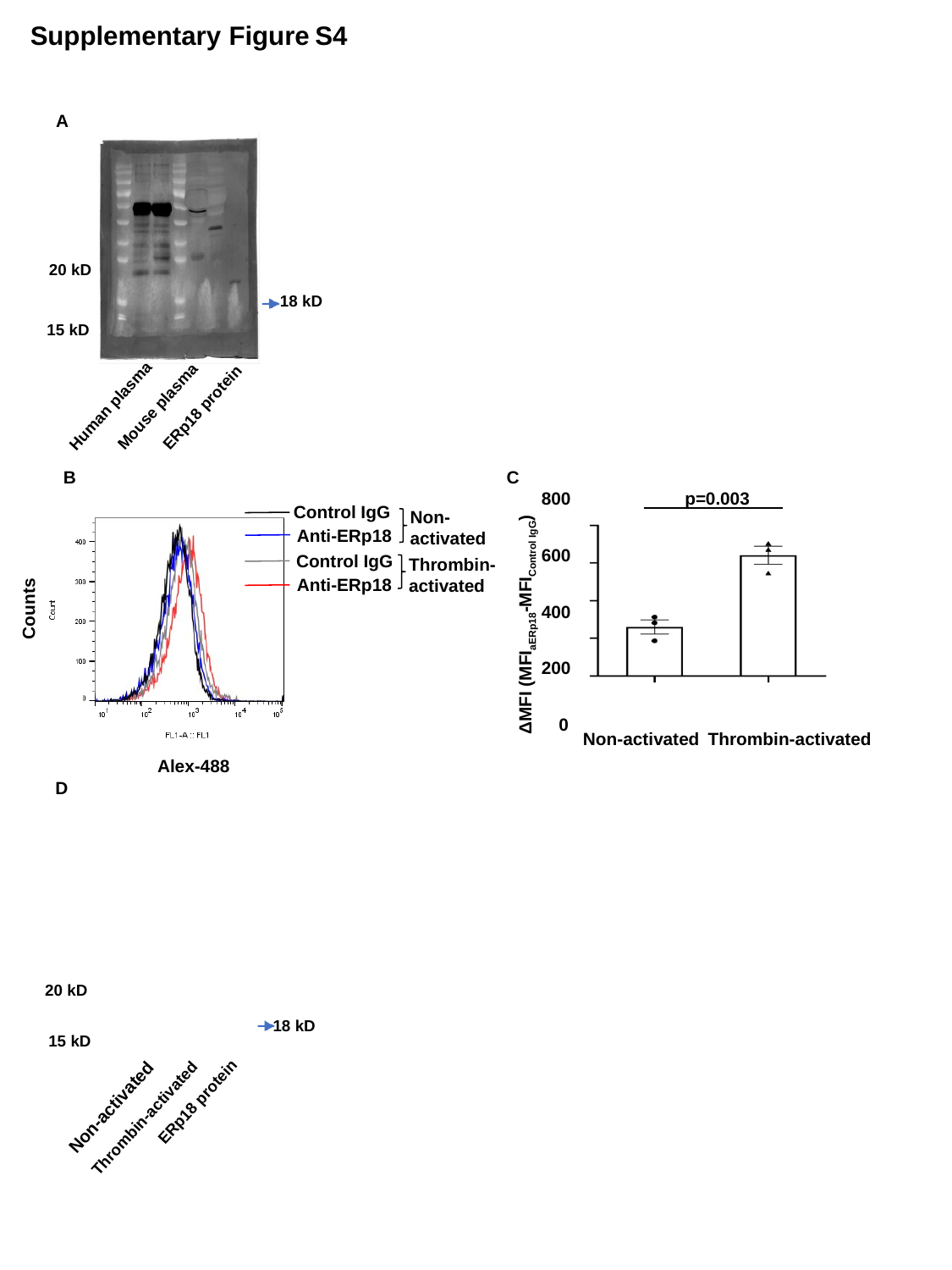

Supplementary Figure S4
A
20 kD
18 kD
15 kD
ERp18 protein
Mouse plasma
Human plasma
B
C
800
p=0.003
600
ΔMFI (MFIaERp18-MFIControl IgG)
400
200
Non-activated
Thrombin-activated
0
 Control IgG
Non-activated
 Anti-ERp18
 Control IgG
 Anti-ERp18
Thrombin-activated
Counts
Alex-488
D
20 kD
18 kD
15 kD
ERp18 protein
Thrombin-activated
Non-activated

## Slide 5
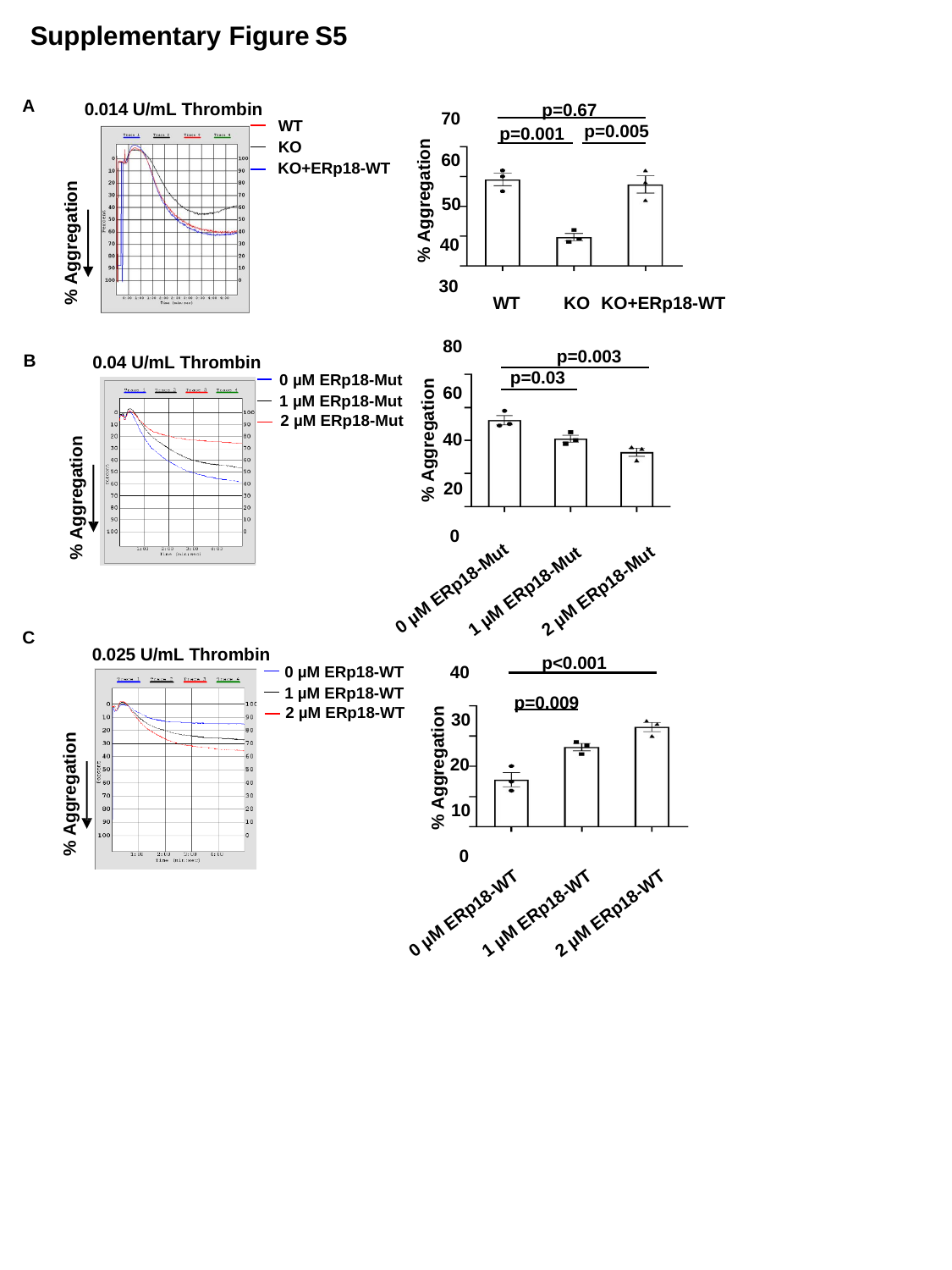

Supplementary Figure S5
A
p=0.67
70
p=0.005
p=0.001
% Aggregation
60
50
40
30
WT
KO
KO+ERp18-WT
0.014 U/mL Thrombin
WT
KO
% Aggregation
KO+ERp18-WT
80
p=0.003
p=0.03
60
% Aggregation
40
20
0
0 µM ERp18-Mut
2 µM ERp18-Mut
1 µM ERp18-Mut
B
0.04 U/mL Thrombin
0 µM ERp18-Mut
1 µM ERp18-Mut
2 µM ERp18-Mut
% Aggregation
C
0.025 U/mL Thrombin
p<0.001
40
p=0.009
30
% Aggregation
20
10
0
2 µM ERp18-WT
0 µM ERp18-WT
1 µM ERp18-WT
0 µM ERp18-WT
1 µM ERp18-WT
2 µM ERp18-WT
% Aggregation

## Slide 6
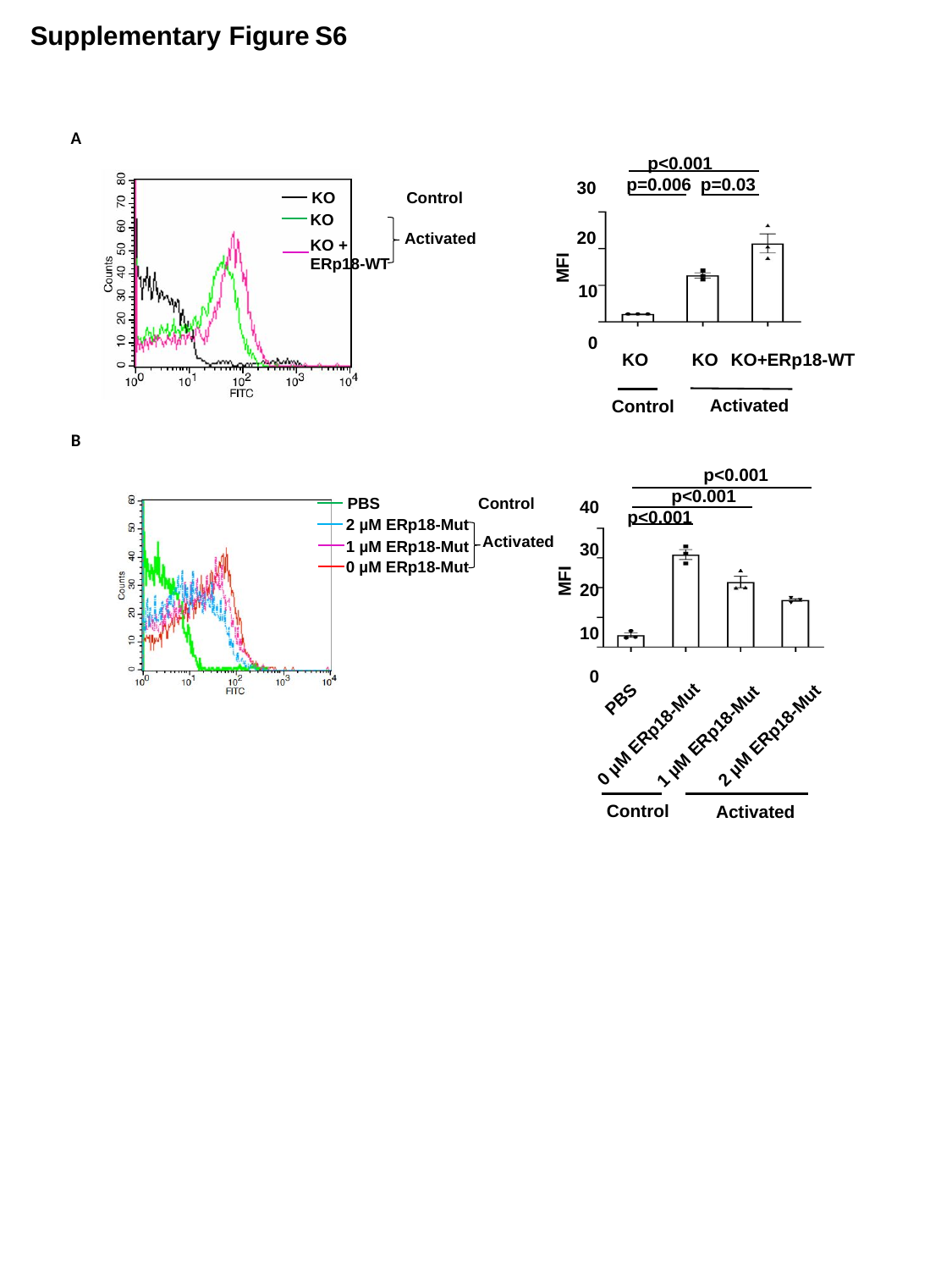

Supplementary Figure S6
A
p<0.001
p=0.006
p=0.03
30
20
MFI
10
0
KO
KO
KO+ERp18-WT
Activated
Control
KO Control
KO
Activated
KO +
ERp18-WT
B
p<0.001
p<0.001
40
30
MFI
20
10
0
PBS
0 µM ERp18-Mut
2 µM ERp18-Mut
1 µM ERp18-Mut
Activated
PBS
2 µM ERp18-Mut
1 µM ERp18-Mut
0 µM ERp18-Mut
Control
p<0.001
Activated
Control

## Slide 7
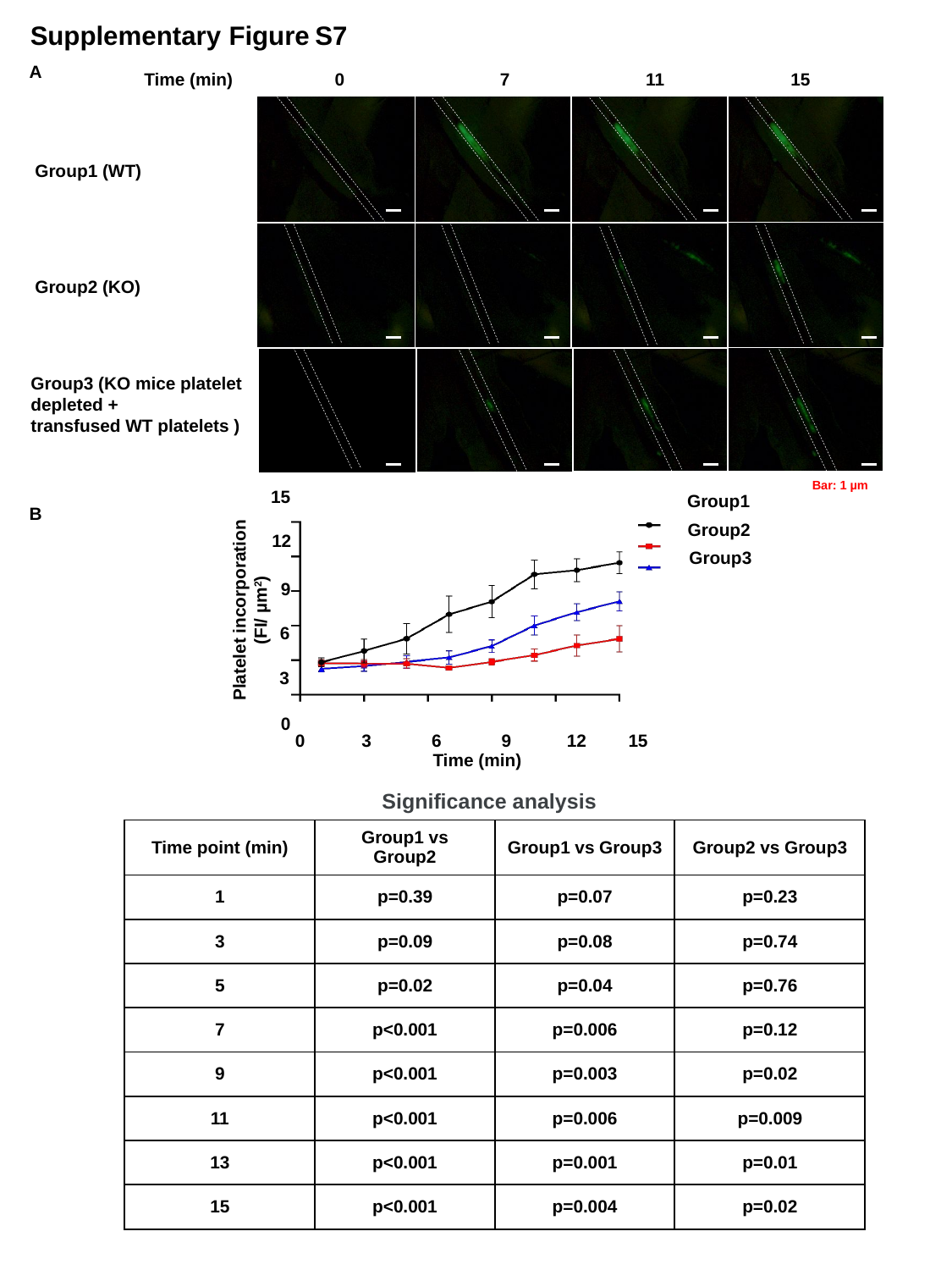

Supplementary Figure S7
A
Time (min) 0 7 11 15
Group1 (WT)
Group2 (KO)
Group3 (KO mice platelet depleted +
transfused WT platelets )
Bar: 1 µm
15
Group1
Group2
12
9
Platelet incorporation
(FI/ µm2)
6
3
0
0
3
6
9
12
15
Time (min)
Group3
B
Significance analysis
| Time point (min) | Group1 vs Group2 | Group1 vs Group3 | Group2 vs Group3 |
| --- | --- | --- | --- |
| 1 | p=0.39 | p=0.07 | p=0.23 |
| 3 | p=0.09 | p=0.08 | p=0.74 |
| 5 | p=0.02 | p=0.04 | p=0.76 |
| 7 | p<0.001 | p=0.006 | p=0.12 |
| 9 | p<0.001 | p=0.003 | p=0.02 |
| 11 | p<0.001 | p=0.006 | p=0.009 |
| 13 | p<0.001 | p=0.001 | p=0.01 |
| 15 | p<0.001 | p=0.004 | p=0.02 |

## Slide 8
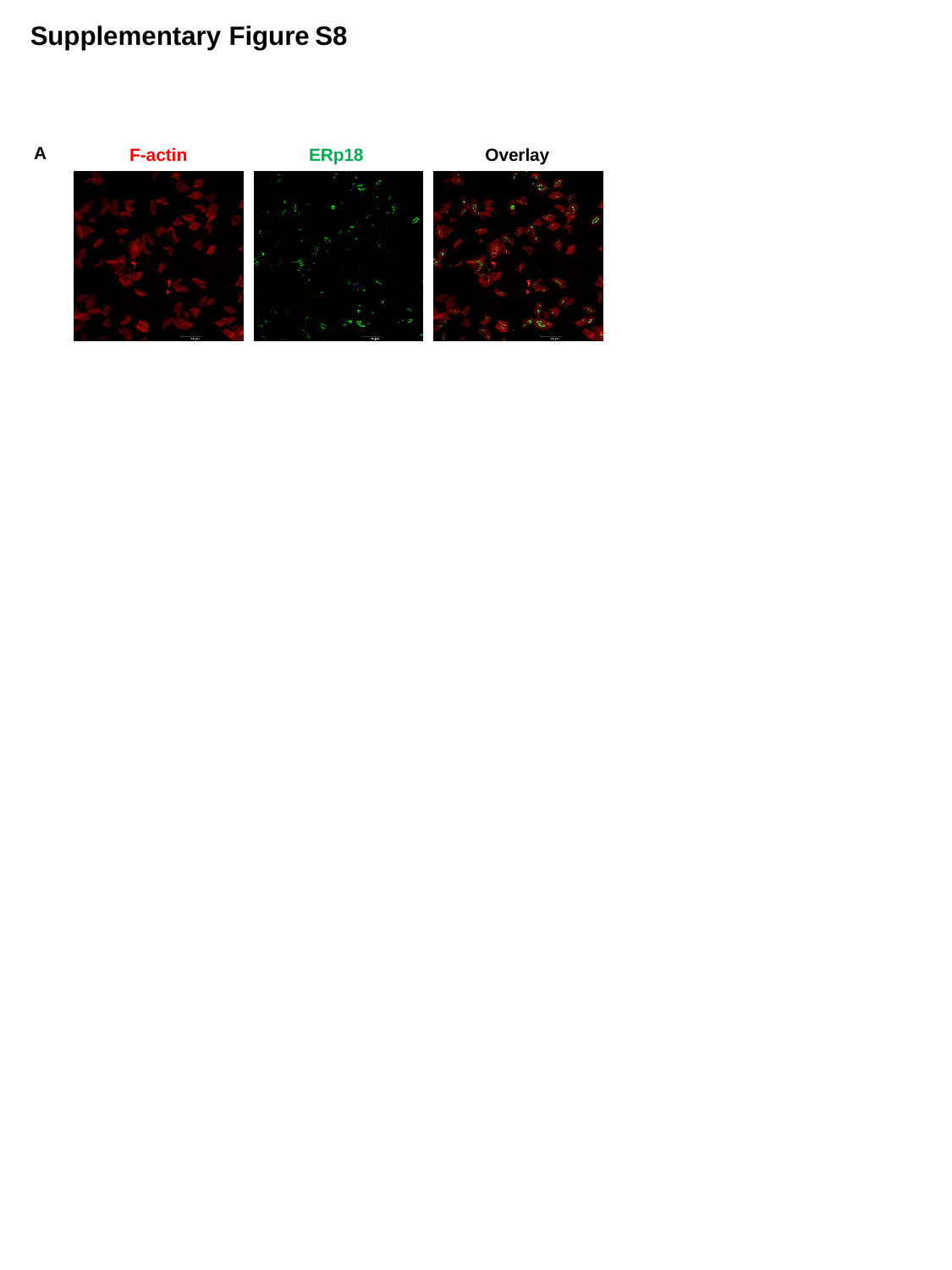

Supplementary Figure S8
A
ERp18
Overlay
F-actin
